# Defects in NK cell immunity of pediatric cancer patients revealed by deep immune profiling

**DOI:** 10.1016/j.isci.2024.110837

**Published:** 2024-08-28

**Authors:** Eleni Syrimi, Naeem Khan, Paul Murray, Carrie Willcox, Tracey Haigh, Benjamin Willcox, Navta Masand, Claire Bowen, Danai B. Dimakou, Jianmin Zuo, Sierra M. Barone, Jonathan M. Irish, Pamela Kearns, Graham S. Taylor

**Affiliations:** 1 Institute of Immunology and Immunotherapy, University of Birmingham, Birmingham, UK; 2 Clinical Immunology Service, Institute of Immunology and Immunotherapy, University of Birmingham, Birmingham, UK; 3 Health research Institute, University of Limerick, Limerick, Ireland; 4 Pathology department, Birmingham Children’s Hospital, Birmingham, UK; 5 Department of Cell & Developmental Biology, Vanderbilt University School of Medicine, Nashville, TN, USA; 6 Department of Pathology, Microbiology and Immunology, Vanderbilt University Medical Center, Nashville, TN, USA; 7 Cancer Research UK Clinical Trials Unit, National Institute for Health Research Birmingham Biomedical Research Centre, Birmingham, UK; 8 Institute of Cancer and Genomic Sciences, University of Birmingham, Birmingham, UK

**Keywords:** Health sciences, Pediatrics, Immunology, Cancer

## Abstract

Systemic immunity plays an important role in cancer immune surveillance and response to therapy, but little is known about the immune status of children with solid cancers. We performed a high-dimensional single-cell analysis of systemic immunity in 50 treatment-naive pediatric cancer patients, comparing them to age-matched healthy children. Children with cancer had a lower frequency of peripheral NK cells, which was not due to tumor sequestration, had lower surface levels of activating receptors and increased levels of the inhibitory NKG2A receptor. Furthermore, the natural killer (NK) cells of pediatric cancer patients were less mature and less cytotoxic when tested *in vitro*. Culture of these NK cells with interleukin-2 restored their cytotoxicity. Collectively, our data show that NK cells in pediatric cancer patients are impaired through multiple mechanisms and identify rational strategies to restore their functionality.

## Highlights

• Systemic NK cell impairment is common in children with cancer
• Peripheral NK cells are less frequent, less mature, and less cytotoxic
• NK cell activating and inhibitory receptors are imbalanced
• *In vitro* culture with IL2 restores NK cell cytotoxicity

## Graphical abstract

**Figure:**
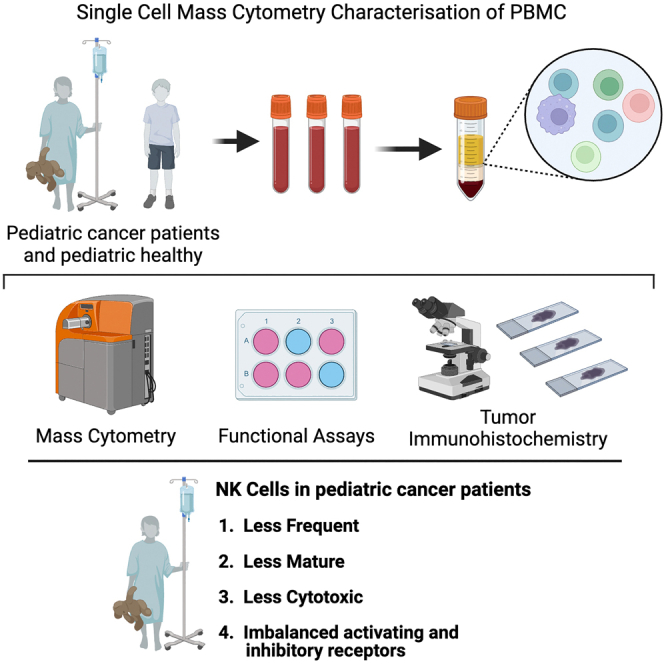

## Introduction

Pediatric cancers are a major cause of childhood mortality.^1^ Current treatments are heavily reliant on cytotoxic chemotherapy and as such are associated with significant acute adverse events. Survivors may suffer multiple long-term treatment-related sequelae and excess mortality due to cardiotoxicity, secondary cancers, and infections.^2^ In addition, current treatments can fail to achieve cure for patients with high risk, metastatic or relapsed disease.^3^ Improving cure rates, while decreasing treatment related sequelae requires new treatment strategies to be developed. One possibility is to harness the immune system to attack the malignant cells. Immunotherapies such as CTLA4 and PD-1 checkpoint inhibitors are effective and now licensed for several adult cancers. However, emerging evidence suggests response rates for checkpoint inhibitors are low for pediatric cancers.^4^

Systemic immune responses are essential for effective immunotherapy^5^ and differences in systemic immunity between patients could contribute to differences in immunotherapy responsiveness.^6^ Current understanding of the immune systems of healthy children is limited and most work has focused on neonates due to the ready availability of cord blood.^6^ A recent longitudinal analysis of immunity in neonates and children up to three months of age shows substantial changes occur in multiple immune cell types that follow a common trajectory.^7^ By three months of age the infant immune system has not yet reached a fully mature state. Substantial changes in the immune system must therefore occur throughout childhood but this process is poorly characterized.

Cancer associated changes in systemic immunity are well documented in adults with cancer. Some of these, for example the increased frequency of immunosuppressive cells like CD4^+^ regulatory T cells (T-regs), are associated with a worse prognosis.^8^ Other changes, such as altered T cell differentiation status, are associated with increased clinical responsiveness to immunotherapy.^9^ Generally, natural killer (NK) cells in adult cancer patients are unaltered in frequency but are less cytotoxic.^10^ Cancer cell-derived soluble NK ligands that bind to receptors on NK-cells, thereby inhibiting target cell recognition, is one possible mechanism.^11^ Cancer associated changes in systemic immunity in children are largely unknown and the extent to which those observed in adult patients or other immune-inhibitory mechanisms operate in children with cancer warrants investigation.

Important differences exist in the biology of cancers that afflict children and adults. Most pediatric cancers have low frequencies of somatic coding mutations^12^ and therefore often lack actionable neoantigens. Furthermore, cancers arising in young children (<5 years old) are often embryonal in origin. A cardinal feature of such tumors, which include hepatoblastoma,^13^ medulloblastoma,^14^ neuroblastoma,^15^ and Wilms tumor^16^ is that they express low or no expression of MHC-class I. While this limits their ability to be controlled by T cells, lack of MHC-I (which delivers an inhibitory signal to NK-cells) should increase tumor sensitivity to NK cell mediated lysis. Indeed, *in vitro* experiments using adult-derived NK cells have shown that established neuroblastoma, glioblastoma,^17^^,^^18^ and hepatoblastoma^13^ cell lines and primary neuroblastoma tumor cells^19^ are all sensitive to NK-mediated lysis. While poor immune cell infiltration into pediatric tumors is a factor thought to limit their immune control, the functional capacity of the NK cell system in pediatric cancer patients remains unknown.

In this study, we used mass cytometry to perform high dimensional single cell analysis of systemic immunity across several childhood solid cancers, comparing patients to age-matched healthy children. We identified important changes in the frequency and phenotype of NK cells and show that some of these changes can be therapeutically targeted. These data not only provide novel insights into pediatric immunity in the context of malignancy but also a rational basis for optimizing the development of immunotherapies for children with cancer.

## Results

### Circulating NK cells are less frequent in pediatric cancer patients

A total of 20 patients were recruited to the initial phase of the study: 19 diagnosed with a range of childhood solid cancers and one patient, C19, with pre-malignant nephroblastomatosis due to the inherited cancer predisposition disorder Wilms tumor aniridia syndrome (WAGR).^20^ Over the same time period 19 age-matched children attending hospital for minor surgeries were recruited as controls. Peripheral blood mononuclear cells (PBMCs) were collected before treatment was commenced and cryopreserved for subsequent analysis. Demographic and clinical information are provided in Table S1 and Figure S1.

**Figure 1:**
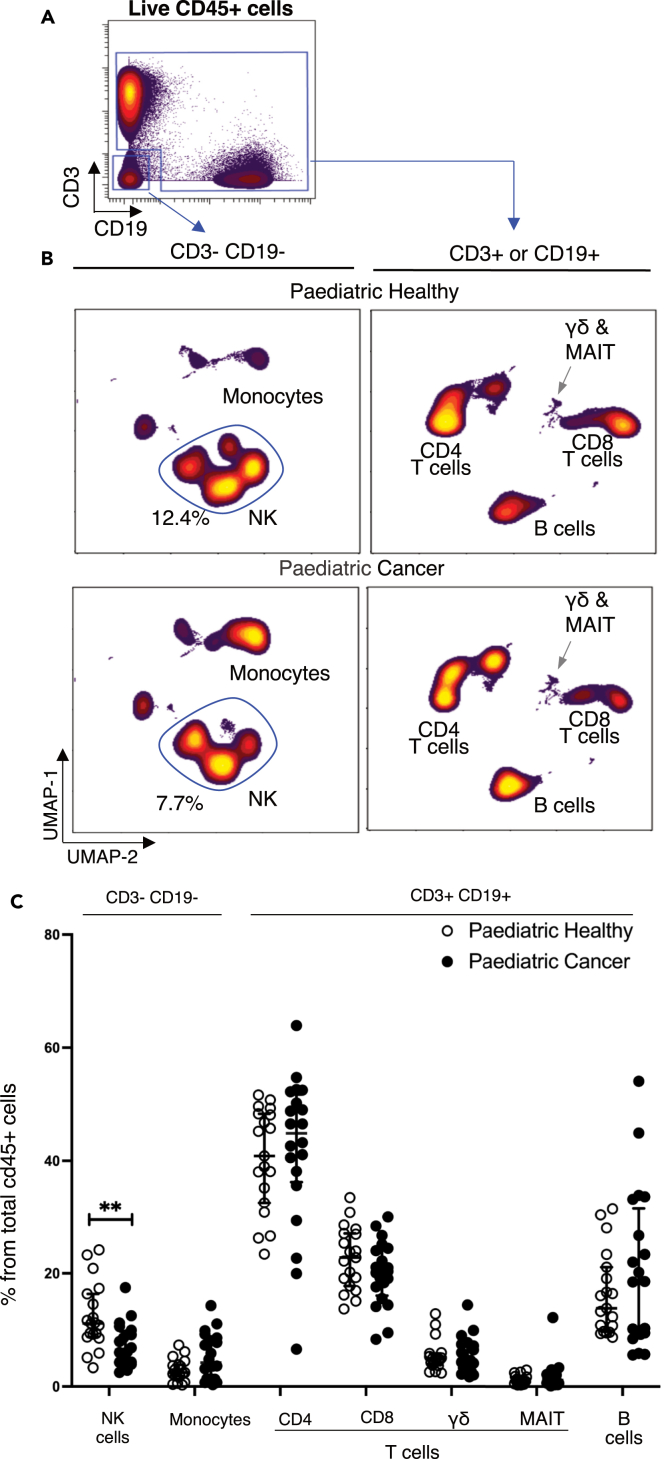
NK and monocyte cell frequencies are altered in pediatric cancer patients (A) Manual gating strategy used to split immune cells into CD3^−^CD19^−^and CD3^+^CD19+subsets. (B) Concatenated FCS files for both pediatric healthy (*n* = 19) and pediatric cancer patients (*n* = 20) were analyzed by UMAP dimensionality reduction. The frequency and identify of main immune subsets is shown. (C) Frequency of the main immune subsets for all healthy children (open symbols) and pediatric cancer patients (closed symbols) expressed as a percentage of total CD45^+^ cells. Error bars show the mean ± 1 standard deviation. *p* values were calculated using Wilcoxon ranked sum tests with false discovery rate at 5% and correction using the Benjamini-Hochberg method. Significant results are indicated: ∗*p* < 0.05, ∗∗*p* < 0.01, ∗∗∗*p* < 0.001.

PBMCs were thawed and analyzed in a single batch by mass cytometry using a 38-marker antibody panel (Table S2). Immune cells were first divided into two separate populations (i. CD3^−^ CD19^−^cells, ii. CD3^+^ or CD19^+^ cells) by manual gating (Figure 1A). Analysis of these two populations by dimensionality reduction revealed differences between patients and controls (Figure 1B). Analyzing cell frequency per individual (Figure 1C) showed pediatric cancer patients had a significantly lower frequency of NK cells (*p* = 0.0035) and a trend for increased monocytes. The frequencies of CD8^+^ T cells, CD4^+^ T cells, gamma-delta T cells, MAIT T cells and B cells were not significantly different between patients and controls (gating strategy shown in Figure S2).

### NK cells in pediatric cancer patients are immature

**Figure 2:**
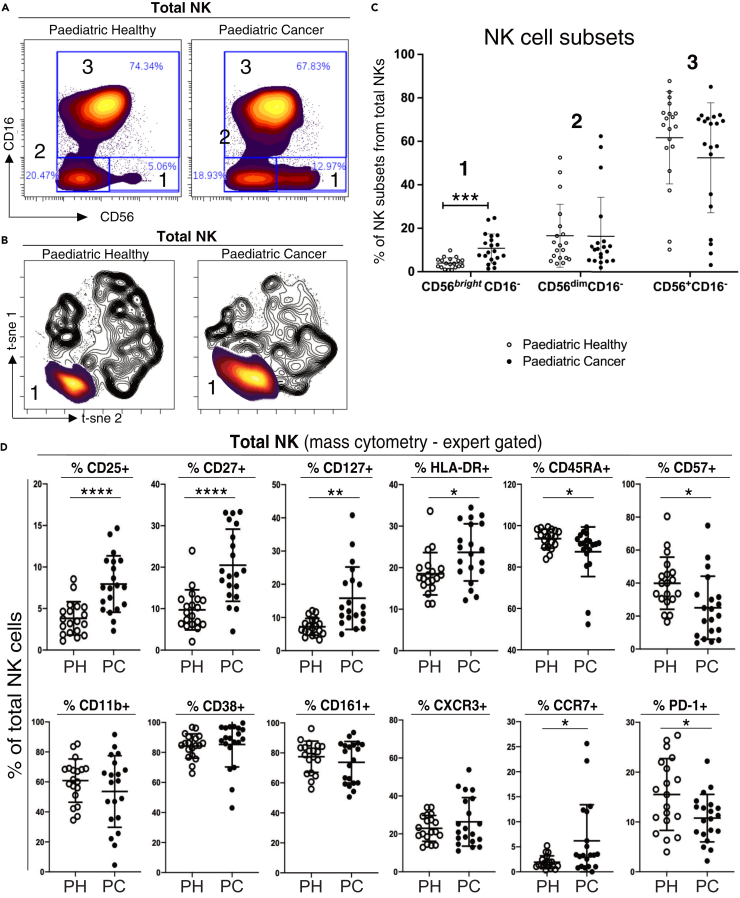
Detailed characterization of NK cells in pediatric cancer patients and controls (A) Biaxial plots of concatenated FCS files from healthy children (*n* = 19) and pediatric cancer patients (*n* = 20). Total NK cells were manually gated using CD16 and CD56 expression to delineate NK cells into three canonical differentiation subsets. In order of maturity: subset 1 (CD56^bright^CD16^-^), 2 (CD56^dim^CD16^-^), 3 (CD56^+^CD16^+^). The proportion of NK cells in each subset is shown on each plot. (B) Heat plots of cell density from subset 1 were overlaid on black and white contour tSNE plots of total NK cells from paediatric healthy donors (left panel) and pediatric cancer patients (right panel). (C) Frequency of each NK subset, expressed as a percentage of total NK cells per individual. (D) Mass cytometry analysis of pediatric cancer patients and paediatric healthy donors showing percentage of total NK cells positive for the indicated markers. The mean ± 1 standard deviation is shown. *p* values were calculated using Wilcoxon ranked sum tests with false discovery rate at 5% and correction using the Benjamini-Hochberg method. Significant results are indicated: ∗*p* < 0.05, ∗∗*p* < 0.01, ∗∗∗*p* < 0.001.

To further investigate the observed differences in NK cell frequency and distribution in the unsupervised analysis (Figures 1B and 1C), we examined the phenotype of NK cells in more detail. After excluding CD3, CD14, and CD19 positive cells and selecting only CD56-positive cells (Figure S2), we manually gated the NK cells into subsets using the classical NK cell markers CD56 and CD16 (Figure 2A). We identified the conventional CD56^bright^CD16^-^ and CD56^dim^CD16^+^ NK cell subsets, generally considered to be immature cytokine producing and mature cytotoxic NK cells, respectively.^21^ We also gated on the unconventional CD56^dim^CD16^-^ NK cells, a subset others have reported to be present in healthy donors and at increased frequency in various diseases.^22^^,^^23^^,^^24^^,^^25^^,^^26^^,^^27^ Analysis of the concatenated cytometry data showed the frequency of immature CD56^bright^CD16^-^ NK cells was 1.4-fold higher in pediatric cancer patients compared to age matched controls (Figure 2B; 12.9% patients, 5.1% controls, *p* = 0.0004). This increase was also apparent when heat plots of the cell density of this NK cell subset were overlaid on black and white contour t-SNE plots of total NK cells (Figure 2C). The frequencies of the other two NK cell subsets (CD56^dim^ CD16^+^ and unconventional CD56^dim^CD16^-^) were not significantly different between patients and controls (Figure 2B).

Examining a broader range of phenotypic markers confirmed that pediatric cancer patients had less mature NK cells (Figure 2D). Patients had a significantly higher proportion of NK cells bearing markers associated with NK immaturity (CD25, CD27, CD127, and HLA-DR)^28^^,^^29^^,^^30^^,^^31^ and a significantly lower proportion of NK cells bearing markers associated with NK maturity (CD45RA and CD57).^32^^,^^33^ There was no difference in the proportion of NK cells expressing markers unrelated to maturity, including the adhesion molecule CD11b,^34^ the adhesion molecule and ectoenzyme CD38^35^ nor CD161, expressed by a distinct subtype of pro-inflammatory NK cells.^36^ There was also no difference in the proportion of NK cells expressing the chemokine receptor CXCR3 that is involved in NK cell recruitment into tumors.^37^ The intensity of PD-1 staining on the NK cells of healthy children and patients was lower than that of T cells (Figure S3) consistent with previous work that found NK cells in adults express low levels of PD1.^38^

**Figure 3:**
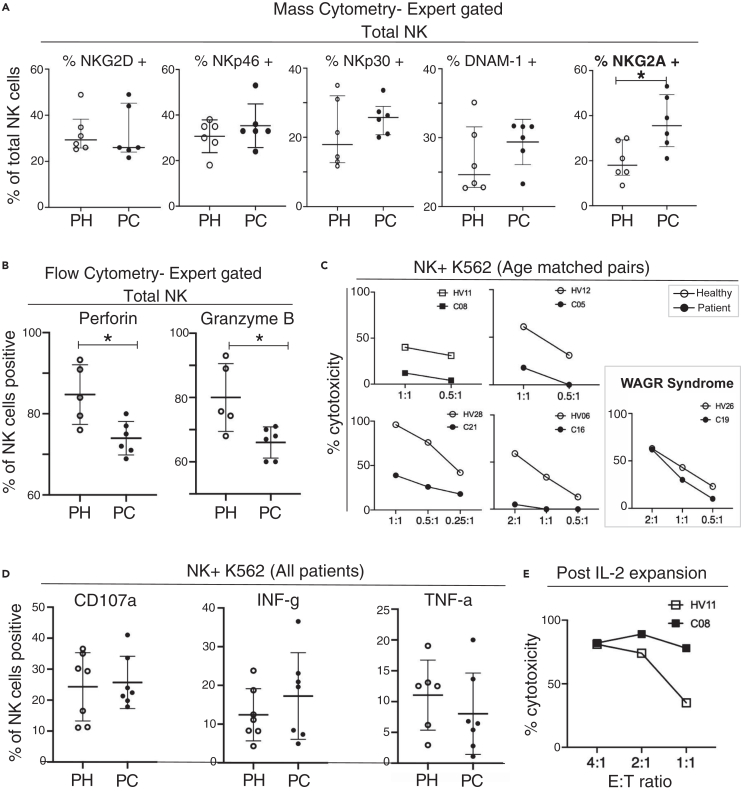
NK cells from pediatric cancer patients show decreased lysis but not decreased degranulation when exposed to K562 target cells (A) Mass cytometry analysis of a subset of the patients and controls shown in Figure 2, now investigating NK receptors. Pediatric healthy donors (PH, *n* = 6); pediatric cancer patients (PC, *n* = 6). (B) Intracellular fluorescent flow cytometry showing percentage of total NK cells positive for perforin or granzyme B. (C) Results of K562 cytotoxicity assays for five patients and five age matched control donors performed at the indicated NK cell to K562 cell effector:target ratios. (D) Flow cytometry analysis of NK cells from seven pediatric healthy donors (PH) and seven pediatric cancer patients (PC) co-incubated with K562 cells. Results include the cancer patients analyzed in C. Degranulation was measured by including a CD107a-specific antibody during the coincubation and cytokine production by intracellular cytokine staining. Error bars show mean ± 1 standard deviation. (E) K562 cytotoxicity assay performed using NK cells from patient C08 and their age matched control HV11 using NK cells expanded *in vitro* with IL-2 for 14 days. E:T indicates the effector: target ratio used. For A, B, and D the results of unpaired t-tests, correcting for multiple comparisons with 5% FDR when necessary, are shown on the figure. ∗*p* < 0.05, ∗∗*p* < 0.01, ∗∗∗*p* < 0.001.

To further explore the phenotype of NK cells in pediatric cancer patients, we developed a separate NK-focused mass cytometry panel (Table S3) focusing on receptors reported to activate or inhibit NK cells.^39^ We used this panel to test six randomly selected patients, comparing them to 6 age matched controls (Figure 3A). The inhibitory receptor NKG2A^39^ was expressed on a significantly higher proportion of NK cells from patients (*p* = 0.0325). There was no difference in the frequency of cells positive for the activating receptors NKG2D, NKp46, NKp30, or DNAM1.^39^ Examining each NK subset in turn, we found more frequent expression of the inhibitory receptor NKG2A^40^ on mature CD56^dim^CD16^+^cytotoxic NK cells (Figure S4, *p* = 0.0018). This significant increase in NKG2A was also detected by unsupervised clustering analysis (Figure S4).

### NK cells from pediatric cancer patients are less cytotoxic in functional assays

Tumor surveillance and clearance by NK cells is mainly dependent upon their cytotoxic functions.^41^ After demonstrating that NK cell subset discrimination by fluorescent and mass cytometry were concordant (Figure S5A) we established a fluorescent antibody panel (Table S4) and measured levels of the intracellular cytotoxic effector molecules perforin and granzyme-B in total NK cells and each of the three NK subsets. Testing samples from six patients and five age matched controls (all selected from the discovery cohort) we found that the total NK cell population in the patient cohort contained significantly lower proportions of cells positive for perforin (*p* = 0.03) and cells positive for granzyme-B (*p* = 0.02) (Figure 3B). This could potentially be explained by the higher frequency of CD56^bright^ CD16^−^ NK cells, which express low levels of perforin and granzymes, in the patients (Figure 2A). However, examining each NK cell subset separately we noted a non-significant trend toward decreased perforin and granzyme B positive cells in the CD56^dim^CD16^+^ and CD56^dim^CD16^-^ subsets (Figure S5B).

We next measured the cytotoxicity of total NK cells in four pediatric cancer patients and age-matched healthy controls, using as target cells the K562 cancer cell line that is widely used to assess NK cell cytotoxicity. We found that NK cells were markedly less cytotoxic in the cancer patients (Figure 3C). In contrast, the NK cells from patient C19, who had WAGR syndrome and pre-malignant nephroblastomatosis rather than an established malignancy, had a similar level of cytotoxicity compared to their age-matched control donor (Figure 3C). To confirm that the decreased killing of K562 cells by NK cells from pediatric cancer patients was due to decreased cytotoxic potential, rather than a decreased ability to degranulate in response to K562 cells, we repeated the assays using alternative readouts of NK cell function. NK cells from the patients and controls displayed similar levels of cell-surface CD107a, a measure of degranulation; they also had similar intracellular levels of interferon-gamma and TNF-a after exposure to K562 cells (Figure 3D).

In adult cancer patients, plasma levels of soluble MICA and ULBP2 proteins are elevated and have been shown to inhibit NK function.^42^ Our pediatric cancer patients had lower plasma concentrations of soluble MICA and ULBP2 than their healthy age matched counterparts excluding this as a possible explanation for the reduced K562 killing we observed (Figure S6). Collectively, our functional assay and cytometry assay data showed that the diminished killing of K562 cells by the pediatric cancer patients’ NK cells was due to a decrease in cytotoxic capacity rather than inefficient target cell recognition. Importantly, we demonstrated that the defect in cytotoxicity could be reversed. Following 14 days *in vitro* culture with IL-2, NK cells from patient C08 killed K562 more efficiently than NK cells from HV11, their age matched counterpart (Figure 3E).

### Analysis of a validation cohort by mass cytometry confirms NK cell perturbations in pediatric cancer patients and reveals imbalanced activating and inhibitory receptor display

**Figure 4:**
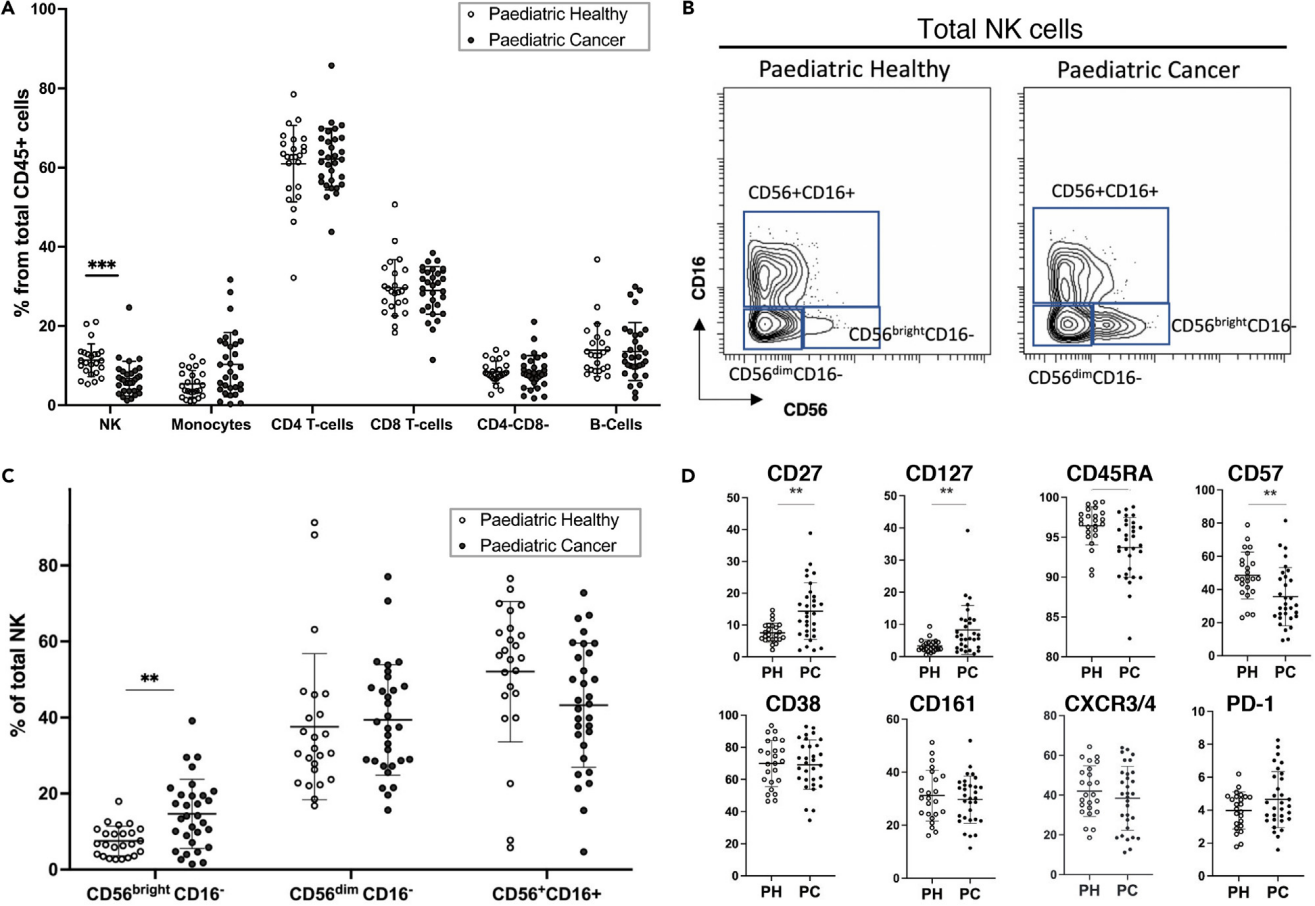
Mass cytometry data from a validation cohort of 31 pediatric cancer (PC) patients and 24 pediatric healthy (PH) donors confirms the NK cell alterations identified in pediatric cancer patients (A) Frequencies of the main immune cell subsets shown at the level of each individual for both pediatric healthy and pediatric cancer patients. (B) Gating strategy for the three NK subsets in pediatric healthy and pediatric cancer patients. (C) Frequency of each NK subset shown for individual for pediatric healthy donors and pediatric cancer patients. (D) Mass cytometry analysis of pediatric cancer patients and pediatric healthy donors showing percentage of total NK cells positive for the indicated markers. The error bars show the mean +/− one standard deviation. *p* values were calculated using Wilcoxon ranked sum tests with false discovery rate at 5% and correction using the Benjamini-Hochberg method. Significant results are indicated: ∗*p* < 0.05, ∗∗*p* < 0.01, ∗∗∗*p* < 0.001.

We collected pre-treatment blood samples from an additional 31 pediatric patients with a range of solid cancers to serve as a validation cohort (Table S5; Figure S1). PBMCs from these patients, and 24 age-matched healthy children, were analyzed by mass cytometry using an NK cell focused antibody panel (Table S6). This panel included antibodies specific for the same core NK cell phenotype and differentiation markers used earlier, as well as additional antibodies to detect key NK cell activating and inhibitory receptors. Manual gating of the major immune cell subsets (Figure S7) showed these patients had the same pattern of NK cell changes we observed in our initial discovery cohort. Compared to children in the control group, the validation cohort of pediatric cancer patients had a significantly lower frequency of peripheral NK cells (controls 11.0%, patients 6.6%, *p* < 0.001, Figure 4A). Calculating the absolute NK cell counts for these patients, 13% of the patients had values below the normal range (Table S7). As seen in the initial cohort, the validation cohort of patients also had a significantly greater proportion of immature CD56^bright^CD16^-^ NK cells (controls 7.2%, patients 18.4%, *p* < 0.01, Figures 4B and 4C). Their NK cells had significantly higher expression of CD27 and CD127 (markers of NK cell immaturity) and significantly lower expression of NK cell maturity markers CD45RA and CD57 (Figure 4D). This lower CD57 expression was not due to the increased frequency of immature CD56bright cells in the total NK population as both CD56dim subsets of NK cells (CD56^dim^ CD16^+^ and CD56^dim^CD16^-^) in cancer patients expressed significantly lower levels of CD57 compared to the same subsets in controls (Figure S8).

Combining the discovery and validation cohorts provided sufficient patient numbers to perform an exploratory subgroup analysis, for patients with Hodgkin lymphoma, neuroblastoma, rhabdomyosarcoma, osteosarcoma, Wilm’s tumor, and Ewing’s sarcoma. Within the limitations of the sample size, our analysis supports the hypothesis that the decreased frequency of total NK cells and the expansion of the CD56^*bright*^CD16^-^ subset are not specific to a particular cancer type and suggests this could be a pan-cancer effect in the pediatric immune system (Figure S9).

**Figure 5:**
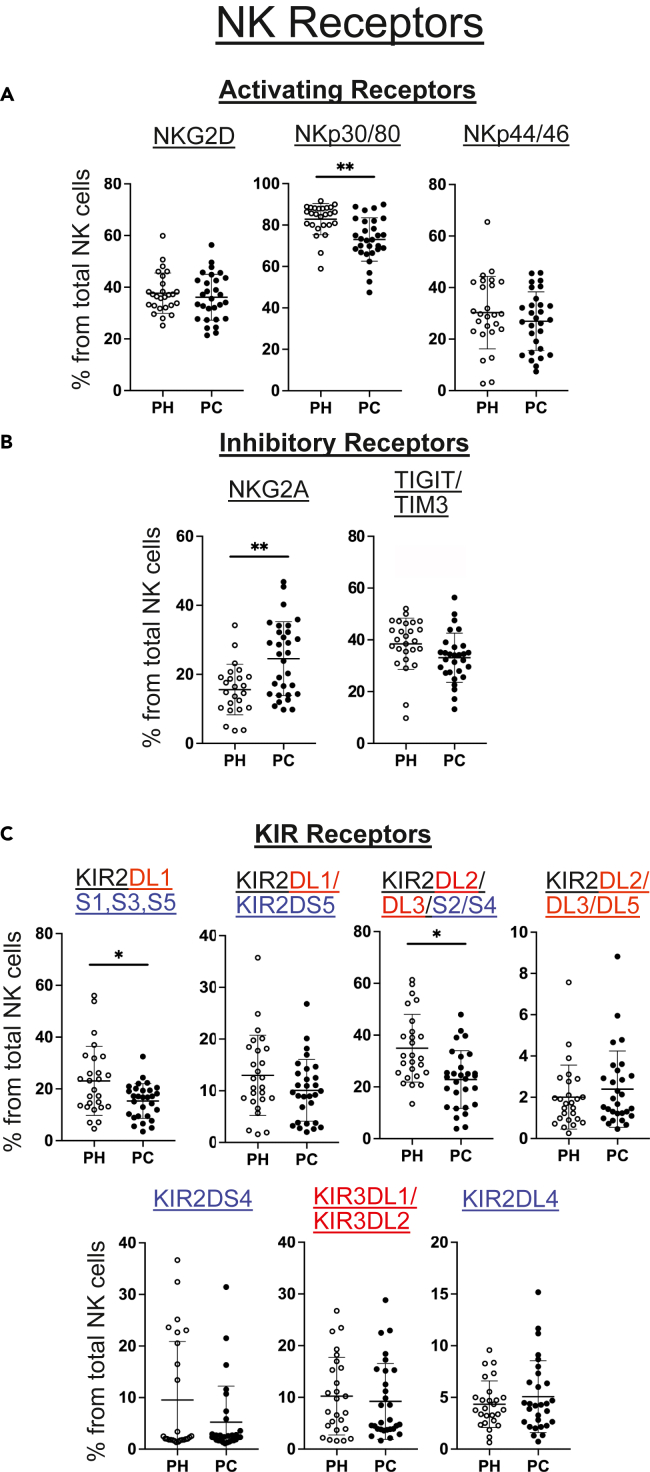
NK receptor analysis reveals an imbalance of activating and inhibitory receptors in pediatric cancer patients (A and B) Mass cytometry data showing the frequency of NK cells in pediatric cancer (PC) patients and pediatric healthy (PH) controls positive for the indicated cell surface receptors. Some markers were co-stained using two antibodies labelled with the same metal isotope. (C) Frequency of NK cells positive for the indicated KIR receptors. Note that some KIR-specific antibodies bind more than one KIR receptor and this is shown on the figure. Cell surface levels of the various NK cell receptors on the total NK cell population was calculated using manual gating and is shown for each pediatric healthy (PH) donor and pediatric cancer (PC) patient. The mean ± 1 standard deviation and results of Wilcoxon ranked sum tests, false discovery rate corrected using the Benjamini-Hochberg method, are also shown. Significant results are indicated: ∗*p* < 0.05, ∗∗*p* < 0.01, ∗∗∗*p* < 0.001. For KIR receptors, activating receptors are shown in blue and inhibitory receptors are shown in red.

Next, we examined surface expression of activating and inhibitory receptors on NK cells from each patient in the validation cohort (Figure 5). Compared to healthy age matched control donors, a smaller proportion of NK cells in the patient cohort expressed the activating receptors NKp30/80 (co-stained on the same mass channel) and a greater proportion NK cells expressed the inhibitory receptor NKG2A.^39^ The median metal intensity of NKG2A staining was also significantly higher on the NKG2A-positive NK cells of the patient cohort (Figure S10). We also measured NK expression of killer cell immunoglobulin-like receptor (KIRs). Flow cytometric analysis of these receptors is complicated by their high sequence homology, which means that currently available antibodies bind multiple KIRs. We therefore used multiple antibodies with overlapping KIR specificities to dissect KIR expression. Compared to the healthy control cohort, a significantly smaller proportion of NK cells from the patients stained with an antibody specific for four KIRs: the inhibitory KIR2DL1^43^ and the activating KIR2DS1, KIR2DS3, and KIR2DS5 molecules^44^ (Figure 5C). A second antibody specific only for KIR2DL1 and KIR2DS5 gave equivalent staining, suggesting this difference was due to lower expression of the activating KIR2DS1 receptor or the activating KIR2DS3 receptor.^45^ Lower frequency of NK cell staining was also observed in the cancer patient cohort using another antibody specific for the inhibitory KIR2DL2 and KIR2DL3 receptors and the activating KIR2DS2 and KIR2DS4 receptors (Figure 5C). However, another antibody specific for KIR2DL2, KIR2DL3, and KIR2DL5 produced equivalent staining (Figure 5C). Collectively, these results support NK cells in pediatric cancer patients having decreased NK cell expression of the activating KIR2DS2 or KIR2DS4 receptors.

Finally, we investigated the NK receptors expressed by each NK subset in pediatric cancer patients and healthy control donors (Figure S11). The pattern of increased frequencies of NK cells expressing inhibitory NKG2A and decreased frequencies of cells expressing activating KIRs that we detected in the total NK population was significant for the cytotoxic CD56^dim^CD16^-^ subset.^40^ Overall, the deep NK cell profiling on the validation cohort confirmed that NK cells in pediatric cancer patients are less mature and revealed additional changes in their receptor expression likely to decrease their function.

### Low blood NK cell frequency in patients is not due to tumor migration

A potential explanation for the decreased frequency of circulatory NK cells in the blood of pediatric cancer patients was that the cells had localized to the tumor. To test this hypothesis, we obtained the diagnostic formalin-fixed, paraffin-embedded tissue biopsies from 17 of the patients (16 cancer cases and the single case of WAGR syndrome) we had analyzed by mass cytometry. Pediatric cancer cells often express NCAM1 (CD56) which is the standard marker used to identify NK cells in the blood.^46^ To determine whether the tumors contained NK cells, we therefore performed two additional stains. First, CD45 to measure overall immune infiltration. Second, CD57 to determine if any infiltrating immune cells were cytotoxic NK cells. Although CD57 cannot definitively identify NK cells because it is also expressed by terminally differentiated T cells, a lack of CD57 positive cells would indicate a lack of cytotoxic NK cells in the tumor.

**Figure 6:**
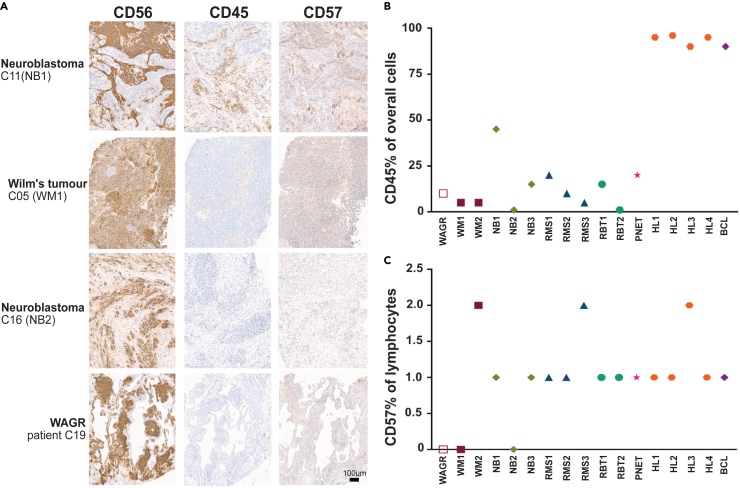
Immunohistochemistry analysis of pediatric tumors (A) Representative images of CD56, CD45 and CD57 staining. Error bar represents 100 um. (B and C) Percentage of CD45^+^ and CD57^+^ cells in the tumors of 16 patients and the nephrablastomatosis lesion from the single patient with WAGR syndrome. WM, Wilms’s tumor; NB, neuroblastoma; RMS, Rhabdomyosarcoma; RBT, Rhabdoid tumor; PNET, primary neuro-ectodermal tumor (embryonal supratentorial tumor); HL, Hodgkin’s lymphoma; BCL, B Cell lymphoma.

As expected, the seven embryonal tumors we tested were positive for CD56 and the five lymphomas we tested were positive for CD45 (representative staining, Figure 6A). For the non-lymphoid tumors, the frequency of CD45-positive infiltrating immune cells, was highly variable (range 1–45%, Figure 6B). Importantly, only a small percentage of infiltrating lymphocytes were positive for CD57 (Figure 6C) suggesting that these tumors contained few cytotoxic NK cells. Therefore, the decreased frequency of circulating total NK cells and CD57^+^ NK cells we observed in the pediatric cancer patients was unlikely to be a consequence of NK cell migration into the tumor.

## Discussion

Cancer is known to alter systemic immunity and while this has been extensively explored in adult cancer patients there is little knowledge of the immune status of children with cancer. Our detailed analysis has revealed that circulating NK cells in young cancer patients with a range of solid malignancies are altered at multiple levels. They are less frequent, less mature, less cytotoxic, and express low levels of activating receptors with concomitant high levels of inhibitory receptors. Importantly, our work provides proof of principle that despite these defects, NK cell cytotoxicity can be restored, potentially allowing them to be targeted therapeutically in cancer therapy.

Our analysis of paired blood and tumor samples suggests that decreased peripheral NK cell frequency in pediatric cancer patients is not caused by NK cell recruitment into the tumor microenvironment but, instead, is due to systemic alteration of the NK cell system. The reason why NK cells have limited access to the tumor microenvironment of pediatric tumors is an important issue. Our phenotyping data show that lack of CXCR3 expression, important for immune cell entry into tumors,^37^ is not the explanation and further mechanistic investigation is a priority for future studies.

NK cells have a short lifetime in the blood, typically two weeks, and must be continuously replenished by the hematopoietic system.^47^ Their low frequency in pediatric cancer patients may therefore reflect a systemic alteration in hematopoesis. This hypothesis is supported by our observation that the NK cell population comprised a greater proportion of immature CD56^bright^ NK cells.^48^ Moreover, this could also reflect a defect in NK cell development and education, which are essential processes for NK cells to be licensed as fully functional self-tolerant effectors that are inhibited from killing human leukocyte antigen (HLA) positive healthy cells.^49^ The self-specific inhibitory KIRs recognize HLA class I molecules and render NK cells hyporesponsive, avoiding potential autoreactivity.^50^ In addition, NKG2A recognizes the non-classical HLA-E molecule and inhibits the function of NK cells.^51^ Therefore, a prerequisite for NK cells to become fully licensed and functional is to express self-specific inhibitory receptors.^52^ This process is followed by several maturation stages which include gradual loss of the NKG2A receptor and acquisition of CD57 and KIR expression.^53^ Our results show that the NK cells of pediatric cancer patients express at least one self-reactive inhibitory receptor for HLA class I, such as KIR or NKG2A, suggesting they have been licensed.^54^ However, the increased proportion of CD56^bright^CD16^-^ NK cells and reduced expression of CD57 on the CD56^dim^ subsets of NK cells of patients clearly demonstrates an alteration in maturity. An increased proportion of circulating immature CD56^bright^ NK cells has been reported in adult cancers and is linked to poor prognosis.^55^ Whether the alterations in NK cell development and maturity we observed in pediatric cancer patients have implications for prognosis would require further evaluation in a larger patient cohort.

The activation of NK cells depends on competing signaling from a wide range of activating and inhibitory receptors.^56^ Regardless of the cause of the NK cell immaturity we observed, our data show that the balance of activating and inhibitory receptors is altered on the NK cells of pediatric cancer patients. Cell surface levels of the inhibitory NK receptor NKG2A^39^ were increased while levels of the activating NK receptors NKp30/80,^57^ KIR2DS1/3, and KIR2DS2/4^45^ were decreased in pediatric cancer patients. This imbalance was particularly apparent for the cytotoxic CD56^dim^ NK subset, which are likely to be the key anti-tumor effector population. Importantly, this increase of NKG2A expression could potentially be therapeutically targeted by, for example, NKG2A blocking antibodies.

NK cells from pediatric cancer patients killed K562 target cells poorly compared to NK cells from control children. One explanation for this difference could be the higher proportion of non-cytotoxic CD56^bright^CD16^-^ cells in the NK cells of patients. However, measuring cell-surface CD107a display showed that the proportion of NK cells that degranulated in response to K562 cells was equivalent for patients and controls. For both groups, only a minority of the perforin- and granzyme-positive NK cells degranulated during the assay. Analysis of NK cells from adults has shown that only a minority of NK cells degranulate in response to K562 cells^58^ and that these cells kill multiple target cells in a sequential manner. This serial killing by a subpopulation of cytotoxic NK cells accounts for the bulk of NK-cell mediated death of K562 cells *in vitro*.^59^^,^^60^^,^^61^ Although serial killing by NK cells results in them gradually losing cytotoxicity, this can be restored when the NK cells receive activating signals via receptors such as NKp30.^62^ The diminished cytotoxicity we observed for NK cells from patients, despite apparently normal levels of degranulation, could therefore be due to their lower expression of activatory receptors failing to deliver the signals needed to sustain serial killing over time.

The mechanisms responsible for the changes in NK cell phenotype and cytotoxicity we observed are currently unknown. Similar to our observations in children with solid cancers, children with acute lymphoblastic leukemia (ALL) have less mature NK cells displaying lower levels of activatory receptors and higher levels of inhibitory receptors.^63^^,^^64^ These changes in NK cells limit their ability to kill ALL blasts.^63^ Regarding the mechanisms involved in driving NK dysregulation in solid pediatric cancers, we showed that these patients did not have raised plasma levels of soluble ULBP2 or soluble MICA, both of which inhibit NK cell function in some adult cancer patients.^42^^,^^65^ Interestingly, the child with pre-malignant nephroblastomatosis that we tested had normal NK-cell mediated cytotoxicity. This represents a rare experiment of nature suggesting that compromised NK cytotoxicity in children with solid cancers may not occur until later in malignant transformation, rather than being an early, enabling event.

By increasing our understanding of the role of NK cell population in the pediatric population, we provide insights into potential new approaches to pediatric cancer immunotherapy. First, using a protocol developed to expand adult NK cells *in vitro* for therapy^66^ we showed it was possible to enhance the cytotoxicity of NK cells from pediatric cancer patients. This clinically validated protocol used IL-2 to stimulate the NK cells. Alternative methods of enhancing NK cell cytotoxicity, such as priming with the cytokine IL-15 that can improve the functionality and cytotoxicity of CD56^bright^ cells, may be more effective.^67^ Second, high expression of the inhibitory NKG2A receptor on the NK cells of pediatric cancer patients provides a rational basis for evaluation of the NKG2A blocking antibody monalizumab,^68^ which has been successfully trialled in some adult cancers.^69^^,^^70^ This approach could be relevant to a wide range of pediatric tumors including neuroblastoma, which expresses high levels of the NKG2A ligand HLA-E.^71^

In summary, our data reveal systemic NK-cell impairment is common in pediatric patients with a wide range of cancers and raises the possibility of targeting the NK cell defects as a therapeutic option. *In vitro* expansion of autologous NK cells or inhibition of NKG2A, for example with monalizumab, are rational treatment strategies that should be considered for pediatric cancer drug development.

### Limitations of the study

Our study has several limitations. The small volumes of blood that could be collected from pediatric cancer patients meant we were able to only test NK cell-mediated cytotoxicity using the K562 cell line. These cells are the standard target for evaluating NK cell-mediated cytotoxicity, allowing our results to be compared to other studies. Although we did not test NK-mediated killing of any pediatric cancer cell lines ourselves, other researchers have shown they can be efficiently killed by NK cells. Small blood sample size also limited our evaluation of NK cell-mediated cytotoxicity enhancement by IL-2. Our data provide an important proof of principle that enhancement is possible but requires confirmation in a larger number of patients. The IL-2 based NK stimulation method we employed was developed for NK cells from adults. The optimal method for pediatric cancer patients requires testing and alternative cytokines, such as IL-15, may be more effective than IL-2. The NK-focused, mass cytometry antibody panel we used to test the validation cohort included some antibody pairs (specific for markers of similar function) labeled with the same metal to extend the range of markers evaluated. Additional experiments will be needed to determine whether levels of one or both markers are altered.

## Resource availability

### Lead contact

Further information and any requests should be directed to and will be fulfilled by the lead contact, Graham Taylor (g.s.taylor@bham.ac.uk).

### Materials availability

This study did not generate new unique reagents.

### Data and code availability

Data reported in this paper will be shared by the lead contact upon request. Mass cytometry data files are available from Mendeley Data (https://doi.org/10.17632/dhfp3wzc77.1). This paper does not report original code. Any additional information required to reanalyse the data reported in this paper is available from the lead contact upon request.

## Acknowledgments

We thank the research nurses at Birmingham Women and Children’s Hospital, Sara-Jane Stanley, Jane Cooper, and Cay Shakespeare, for their help and support recruiting to the TRICICL study. Matt Wakeman, Birmingham and Women’s Hospital Pathology department, assisted with immunohistochemisty staining. We thank the patients, their families, and all healthy volunteers for their participation in the study.

Funding: This work was funded by the Cancer Research UK Birmingham Centre and Birmingham Children’s Hospital Charity (grant: BCHRF479). This work was supported in part by the 10.13039/501100008530European Regional Development Fund (no. CZ.02.1.01/0.0/0.0/16_019/0000868) and 10.13039/100000002NIH / 10.13039/100000054NCI grant R01 CA226833 (J.M.I. and S.M.L.).

## Author contributions

E.S., P.K., and G.T. conceived and designed the study. Samples were collected and processed by E.S., T.H., N.M., and G.T. Data were generated by E.S., N.K., C.W., and G.T. Data analysis and interpretation were performed by E.S., J.I., S.B., C.W., B.W., J.Z., P.M., P.K., and G.T. Manuscript writing was performed by E.S., J.I., P.K., and G.T. Analysis of tissue specimens was performed by C.B. and E.S. The final version of the manuscript was approved by all authors who are all accountable for the work.

## Declaration of interests

The authors declare no competing interests.

## STAR★Methods

### Key resources table

**Table:** 

REAGENT or RESOURCE	SOURCE	IDENTIFIER
**Antibodies**		

Anti-CD45 (clone H130, purified)	Biolegend	Cat#304002
Anti-CD31 (clone WM59, purified)	Biolegend	Cat#303102
Anti-CD57 (clone HCD57, conjugated to 142Nd)	Standard BioTools	Cat#3142007B
Anti-CD38 (clone HIT2, purified)	Biolegend	Cat#303502
Anti-CD8 (clone SK1, purified)	Biolegend	Cat#344727
Anti-CD4 (clone RPA-T4, conjugated to 145Nd)	Standard BioTools	Cat#3145001B
Anti-IgD (clone IA6-2, conjugated to 146Nd)	Standard BioTools	Cat#3146005B
Anti-CXCR3 (clone G025H7, purified)	Biolegend	Cat#353702
Anti-CD16 (clone 3G8, conjugated to 148Nd)	Standard BioTools	Cat#3148004B
Anti CD127 (clone A019D5, conjugated to 149Sm)	Standard BioTools	Cat#3149011B
Anti-OX40 (clone ACT35, conjugated to 150Nd)	Standard BioTools	Cat#3150023B
Anti-CCR6 (clone G034E3, purified)	Biolegend	Cat#353402
Anti-TCR-γδ (clone 11F2, conjugated to 152Sm)	Standard BioTools	Cat#3152008B
Anti-CCR4 (clone L291H4, purified)	Biolegend	Cat# 359402
Anti-CD73 (clone AD2, purified)	Biolegend	Cat# 344002
Anti-PD1 (clone EH12.2H7, conjugated to 155Gd)	Standard BioTools	Cat#3155009B
Anti-CD45RA (clone HI100, purified)	Biolegend	Cat#304102
Anti-CD33 (clone WM53, conjugated to 158Gd)	Standard BioTools	Cat#3158001B
Anti-CD161 (clone HP-3G10, conjugated to 159Tb)	Standard BioTools	Cat#3159004B
Anti-CD39 (clone A1, conjugated to 160Gd)	Standard BioTools	Cat#3160004B
Anti-ICOS (clone C398.4A, purified)	Biolegend	Cat# 313502
Anti-CD27 (clone L128 conjugated to 167Er)	Standard BioTools	Cat#3167006B
Anti-CD56 (clone NCAM16.2, conjugated to 163Dy)	Standard BioTools	Cat#3163007B
Anti-CD95 (clone DX2, conjugated to 164Dy)	Standard BioTools	Cat#3164008B
Anti-CD19 (clone HIB19, conjugated to 165Ho)	Standard BioTools	Cat#3165025B
Anti-CD24 (clone ML5, conjugated to 166Er)	Standard BioTools	Cat#3166007B
Anti-CCR7 (clone G043H7, purified)	Biolegend	Cat#353202
Anti-CXCR5 (clone J252D4, purified)	Biolegend	Cat#356902
Anti-CD25 (clone 2A3 conjugated to 169Tm)	Standard BioTools	Cat#3169003B
Anti-CD123 (clone 6H6, purified)	Biolegend	Cat# 306002
Anti-CD5 (clone UCHT2, purified)	Biolegend	Cat# 300602
Anti-CD11c (clone 3.9, purified)	Biolegend	Cat#301602
Anti-CD3 (clone UCHT1, purified)	Biolegend	Cat#300402
Anti-HLA-DR (clone L243, conjugated to 174Yb)	Standard BioTools	Cat# 3174001B
Anti-CD14 (clone M5E2, conjugated to 175Lu)	Standard BioTools	Cat# 3175015B
Anti-TCRva7.2 (clone 3C10, purified)	Biolegend	Cat#351702
Anti-CD11b (clone ICRF44, conjugated to 209Bi)	Standard BioTools	Cat#3209003B
Anti-TCR gamma-delta 2 (clone 123R3, purified)	Miltenyi	Custom request
Anti-CX3CR1 (clone 2A91, purified)	Biolegend	Cat#341602
Anti-TIGIT (clone A15153G, purified)	Biolegend	Cat#372702
Anti-CD45RA (clone HI100, purified)	Biolegend	Cat#304102
Anti-NKp30 (clone Z25, conjugated to 159Tb)	Standard BioTools	Cat#3159017B
Anti-TCR-delta 1 (clone REA173, purified)	Miltenyi	Cat#130-122-285
Anti-NKp46 (clone BAB281, conjugated to 162Dy)	Standard BioTools	Cat#3162021B
Anti-NKG2C-PE (clone 134591)	R&D Systems	Cat#FAB138P
Anti-PE (clone PE001, conjugated to 165Ho)	Standard BioTools	Cat#3165015B
Anti-NKG2D (clone ON72, conjugated to 166Er)	Standard BioTools	Cat#3166016B
Anti-NKG2A (clone Z199, conjugated to 169Tm)	Standard BioTools	Cat#3169013B
Anti-CD161 (clone HP-3G10, purified)	Biolegend	Cat#339902
Anti-DNAM (clone DX11, conjugated to 171Yb)	Standard BioTools	Cat#3171013B
Anti-CD3-e450 (clone SK7)	Thermo Fisher	Cat#48-0036-42
Anti-CD56-APC (clone CMSSB)	Thermo Fisher	Cat# **17-0567-42**
Anti-CD16-PE-Cy7 (clone eBioCB16)	Thermo Fisher	Cat# **25-0168-42**
Anti-CD57-PerCP-Cy5.5 (clone QA17A04)	Biolegend	Cat#393312
Anti-CD107a-FITC (clone H4A3)	Biolegend	Cat#328606
Anti-TNFa-AF700 (clone MAB11)	Biolegend	Cat# 502928
Anti-IFNg-PE (clone 4S.B3)	Biolegend	Cat#502510
Anti-CD19-APC-Cy7 (clone HIB19)	Biolegend	Cat#302218
Anti-CD14-APC-Cy7 (clone 63D3)	Biolegend	Cat#367108
Anti-Perforin-PE-Cy7 (clone dG9)	Thermo Fisher	Cat#25-994-42
Anti-GranzymeB-PE-Texas-Red (clone GB11)	Invitrogen	Cat#10558363
Anti-CCR2 (clone K036C2, purified)	Biolegend	Cat#357202
Anti-CD57 (clone HNK-1, purified)	Biolegend	Cat# 359602
Anti-CD36 (clone 5-271, purified)	Biolegend	Cat# 336202
Anti-CD69 (clone FN50, conjugated to 144Nd)	Standard BioTools	Cat#3144018B
Anti-KIR2DL1/S1/S3/S5 (clone HP-MA4, purified)	Biolegend	Cat#339502
Anti-CXCR1 (clone 42705, purified)	R&D Biosystems	Cat#MAB330
Anti-CXCR2 (clone 5E8/CXCR2, conjugated to 147Sm)	Standard BioTools	Cat#3147010B
Anti-CD14 (Clone RMO52, conjugated to 148Nd)	Standard BioTools	Cat#3148010B
Anti-CD25 (clone 2A3, conjugated to 149Sm)	Standard BioTools	Cat#3149010B
Anti-CD27 (clone LG.3A10, conjugated to 150Nd)	Standard BioTools	Cat#3150017B
Anti-KIR2DL1/S5 (clone 143211, purified)	R&D Biosystems	Cat# MAB1844
Anti-ILT1 (clone 24, purified)	Biolegend	Cat#337902
Anti-CXCR5 (clone RF8B2, conjugated to 153Eu)	Standard BioTools	Cat#3153020B
Anti-TIGIT (clone MBSA43, conjugated to 154Sm)	Standard BioTools	Cat#3154016B
Anti-TIM3 (clone F38-2E2, conjugated to 154Sm)	Standard BioTools	Cat#3154010B
Anti-CD56 (clone B159, conjugated to 155Gd)	Standard BioTools	Cat#3155008B
Anti-ILT2 (clone GHI/75, conjugated to 156Gd)	Standard BioTools	Cat#3156020B
Anti-ILT2/LIR6 (clone 586326, purified)	R&D Biosystems	Cat#MAB30851
Anti-NKp80 (clone 239127, purified)	R&D Biosystems	Cat#MAB1900
Anti-CXCR6 (clone K041E5, conjugated to 160Gd)	Standard BioTools	Cat#3160016B
Anti-ILT3 (clone ZM4.1, purified)	Biolegend	Cat#333002
Anti-ILT5 (clone 222821, purified)	R&D Biosystems	Cat#MAB1806
Anti-ILT4 (clone 42D1, conjugated to 161Dy)	Standard BioTools	Cat#3161019B
Anti-NKp44 (clone P44-8, purified)	Biolegend	Cat#325102
Anti-CXCR4 (clone 12G5, purified)	Biolegend	Cat#306502
Anti-CXCR3 (clone G025H7, conjugated to 163Dy)	Standard BioTools	Cat#3163004B
Anti-CD161 (clone HP3G10, conjugated to 164Dy)	Standard BioTools	Cat#3164009B
Anti-KIR2DS4 (clone 179315, purified)	R&D Biosystems	Cat#MAB1847
Anti-NKG2D (clone ON72, conjugated to 166Er)	Standard BioTools	Cat#3166016B
Anti-KIR3DL1 (clone DX9, conjugated to 167Er)	Standard BioTools	Cat#3167013B
Anti-KIR3DL2 (clone 539304, purified)	R&D Biosystems	Cat# MAB2878
Anti-CD127 (clone A019D5, conjugated to 168Er)	Standard BioTools	Cat#3168017B
Anti-NKG2A (clone Z199, conjugated to 169Tm)	Standard BioTools	Cat#3169013B
Anti-CD122 (clone Tu27, conjugated to 170Er)	Standard BioTools	Cat#3170004B
Anti-CD226 (clone DX11, conjugated to 171Yb)	Standard BioTools	Cat#3171013B
Anti-CX3CR1 (clone 2A9-1, purified)	Biolegend	Cat#341602
Anti-KIR2DL2/L3 (clone DX27, conjugated to 173Yb)	Standard BioTools	Cat#3173010B
Anti-KIR2DL5 (clone UP-R1, purified)	Miltenyi	Cat#130-096-199
Anti-CD94 (clone HP-3D9, conjugated to 174Yb)	Standard BioTools	Cat#3174015B
Anti-PD1 (clone EH12.2H7, conjugated to 175Lu)	Standard BioTools	Cat#3175008B
Anti-KIR2DL4 (clone 181703, purified)	R&D Biosystems	Cat#MAB2238
Anti-KIR2DL2/L3/S2/S4 (clone 180704, purified)	R&D Biosystems	Cat#MAB1848
Anti-CD16 (clone 3G8, conjugated to 209Bi)	Standard BioTools	Cat#3209002B

**Biological samples**		

PBMC, serum and plasma from healthy children.	N/A	N/A

**Chemicals, peptides, and recombinant proteins**		

Maxpar X8 Antibody Labelling kits (various isotopes)	Fluidigm	various
Maxpar MCP9 Antibody Labelling kits (various isotopes)	Fluidigm	various
EQ four element calibration beads	Fluidigm	Cat#201078
Trustain Fc receptor blocking solution	Biolegend	Cat#422302
Cell-ID iridium intercalator	Fluidigm	Cat#201192B
Cell Staining Media (CSM, phosphate buffered saline + 0.5% foetal bovine serum + 0.02% sodium azide)	Prepared in house	N/A

**Deposited data**		

Mass cytometry datafiles (antibody panels 1 and 2).	Mendeley Data	https://doi.org/10.17632/dhfp3wzc77.1

**Software and algorithms**		

Prism	Graphpad	
Cytobank Cytometry Analysis Software	Cytobank	
R (version 3.5.3)	www.R-project.org	

**Other**		

Helios Mass Cytometer	Fluidigm	N/A
LSRFortessa X-20 Cytometer	Beckton Dickinson	N/A

### Experimental model and study participant details

#### Ethical approval and consent

All patient and healthy volunteer samples were obtained Birmingham Children’s Hospital as part of a Health Research Authority approved study (TrICICL) reviewed and approved by South of Birmingham Research Ethics Committee (REC: 17/WM/0453, IRAS: 233593). The study was performed in accordance with the declaration of Helsinki and written informed consent was obtained from all participants or their legal guardians.

#### Study participants and sample collection

Blood samples from a total of 84 children (age range 4 months to 16 years, male:female ratio 1.2:1) were analysed in this study. Of these children, 51 had been diagnosed with cancer and 33 had not and served as healthy controls). Every individual’s age, sex and, where applicable, cancer diagnosis is provided in Tables S1 and S5 (for the discovery and validation cohorts respectively). There was no significant difference in age between the healthy children and children with cancer (Figure S1). Analysis of sex-based differences was not performed due to sample size. Blood samples were collected before cancer treatment was started.

### Method details

#### Sample processing and storage

Peripheral blood samples from pediatric cancer and healthy patients were collected in EDTA tubes, stored at room temperature, and were processed within 24 hours. Peripheral blood mononuclear cells (PBMCs) were isolated from blood samples using SepMate tubes (Stemcell technology) as per the manufacturer’s protocol. PBMCs were cryopreserved in medium containing DMSO^72^ before transfer to liquid nitrogen for long-term storage.

#### Clinical laboratory data

The absolute lymphocyte count for patients recruited to the validation cohort was determined by the Birmingham Children’s Hospital’s clinical laboratory). The absolute count of NK cells was determined by the University of Birmingham Clinical Immunology Service using Trucount tubes and a FACSCanto-II cytometer. Pediatric reference values were obtained from previously published data.^73^

#### Mass cytometry (CyTOF) panel

Two panels of antibodies were designed and used for deep immunophenotypic analysis (Tables S2 and S3). Antibodies were purchased pre-conjugated from Standard BioTools or unconjugated from Biolegend and conjugated in house using the Maxpar antibody labelling kit from Standard BioTools following manufacturer’s protocol.

#### Staining of cells for mass cytometry

Cryopreserved PBMC were recovered into cell culture media (RPMI, 10% Fetal Bovine Serum, penicillin 50U/ml and streptomycin 50U/ml) and washed once. Equal numbers of viable cells were transferred into FACS tubes for barcoding with CD45 specific antibodies conjugated with different metal isotopes. PBMCs from cancer patients, pediatric healthy donors and adult volunteers were barcoded using a batch randomisation scheme to avoid bias. After 20 minutes incubation with the barcoding antibody, cells were washed and then multiple samples (each stained with a different barcoding antibody) were combined into a single tube. Also included in the experiments were buffy coat PBMCs, also barcoded with each of the different metals, to allow technical variability to be assessed.

Following barcoding, cells were stained with phenotyping antibodies. A master-mix of all 36 phenotyping antibodies was prepared by adding the appropriate pre-tested dilutions into filtered cell stain media (CSM - phosphate buffered saline + 0.5% Foetal Bovine Serum + 0.02% sodium azide). After 30 minutes incubation, live dead rhodium stain (Standard BioTools) was added. The samples were washed twice then fixed overnight with freshly prepared 1.6% formaldehyde (Thermo Scientific). The following day, cells were incubated with iridium intercalator (Standard BioTools) solution for one hour. Cells were washed once in CSM and twice in deionised water. Cells were resuspended in deionised water spiked with Four Element Calibration Beads (Standard BioTools) and then filtered through a 70μm cell strainer. Data acquisition was performed using a Standard BioTools Helios mass cytometer with the HT injector at an event rate of less than 500 per second. Data normalisation was performed on raw FCS files using the Helios data acquisition software (Standard BioTools). Normalised data were uploaded to Cytobank for further analysis including de-barcoding, manual, and automated analysis.

#### NK cytotoxicity assay

Cytotoxicity of NK cells was determined by measuring their ability to kill the erythroleukemia cell line K562. PBMCs were recovered and incubated overnight in culture media (RPMI, 10% Foetal Bovine Serum, Penicillin 50U/ ml and streptomycin 50U/ml) plus 200IU/ml IL-2 (Peprotech). Cells from each donor were co-cultured with CFSE labelled K562 (CFSE tracking kit, Biolegend) correcting for differences in NK cell frequency in the PBMCs so the effector to target ratios were defined. Cells were cultured at 37^o^C in cell culture media (RPMI, 10% foetal bovine serum, penicillin 50U/ ml and streptomycin 50U/ml). The following day cells were washed once with PBS and incubated for 10 minutes with efluor 780 live dead stain (ebioscience). Counting beads (ebioscience) were added to the sample and data acquired using an LSR-II flow cytometer. Cytotoxicity was calculated using the formula cytotoxicity= [(expected live target cells - live target cells)/ expected live target cells] x100.

#### NK cell degranulation assay

NK cell degranulation was measured similarly to NK cytotoxicity. PBMCs were recovered from cryopreservation and incubated overnight in IL-2 containing media as described above. PBMCs were then co-cultured with K562 cells at defined NK:K562 cell ratios. Anti-CD107a-FITC antibody (Biolegend) was added at the beginning of the assay at 1ul/well. After one hour, monensin and brefeldin A (Biolegend) were added to the assay as per manufacturer’s protocol. At the end of the incubation cells were stained with fluorophore-conjugated antibodies specific for CD3, CD16, CD56 and CD57. Cells were then fixed and permeabilized using the eBioscience fixation and permeabilization kit and intracellular staining for INF-g and TNF-a performed. Cells were then acquired on an LSR-II flow cytometer and data analysed in Cytobank.

#### NK expansion assay

A minimum of 500,000 PBMCs were resuspended in culture media consisted of RPMI/5% human albumin serum/500IU/ml IL-2/1% Penicillin-streptomycin solution.^66^ Cells were placed in a 48 well plate at a density of 500,000 cells per well. NK cells were cultured *in vitro* for 12-14 days with media replacement every three days. Cytotoxicity of the cultured NK cells was then assessed as described above.

#### Immunohistochemistry

FFPE sections of patients’ tumours were stained at Birmingham Children’s Hospital pathology department for CD45, CD56 and CD57, using lymphoid tissue as quality control for the staining. Slides were scanned and analysed using the Aperio eSlide manager software. Slide staining was validated and scored by a consultant pathologist.

### Quantification and statistical analysis

#### Mass cytometry data analysis

Details of the staining protocol is provided in supplementary methods. Bead normalised data were analysed in Cytobank (Beckman Coulter Inc.). Samples from the discovery cohort were debarcoded, down sampled to 4751 cells per sample and all FCS files from patients or from healthy donors were concatenated for further analysis. Gating strategies are provided in Figures 1 and S2. Dimensionality reduction and clustering were performed in Cytobank using the viSNE implementation of tSNE and FlowSOM respectively. UMAP was performed using the UWOT package and robust linear modelling performed using the MASS package in R version 3.5.3. Marker enrichment modelling v3.0 was performed in R using code downloaded from GitHub. Additional statistical tests, multiple t-test (correcting for multiple comparisons using the Benjamini, Krieger and Yekuteli two stage step up method with 5% FDR) or unpaired Mann-Whitney test, were performed in GraphPad prism v8.

Similarly, samples for the validation cohort were taken at the time of diagnosis and before any treatment was administrated. Samples were debarcoded, down sampled to 11,549 cells per sample and all fcs files from patients or healthy donors concatenated for further analysis for visualisations purposes. Dimensionality reduction and clustering were performed in Cytobank using the viSNE implementation of tSNE and FlowSOM respectively. Additional statistical tests, multiple t-test (correcting for multiple comparisons using the Benjamini, Krieger and Yekuteli two stage step up method with 5% FDR) or unpaired Mann-Whitney test, were performed in GraphPad prism v8.

#### Data analysis and statistical testing

For the pediatric cancer cohorts GraphPad Prism v8 (GraphPad Software) was used for statistical testing for all experiments. The detail of each statistical test is provided in each figure legend. P values were considered to be significant and reported in the text when p<0.05 and when significance was retained after correcting for multiple comparisons by false discovery rate.
