# Supplementary material for: Defects in NK cell immunity of pediatric cancer patients revealed by deep immune profiling
Source: iScience. 2024 Aug 28;27(9):110837. doi: 10.1016/j.isci.2024.110837 (PMC11416690; doi:10.1016/j.isci.2024.110837)
Supplement: Document S1. Figures S1–S1 and Tables S1–S7 [file mmc1.pdf]

**iScience, Volume *27***

## Supplemental information

# Defects in NK cell immunity of pediatric cancer patients revealed by deep immune profiling

**Eleni Syrimi, Naeem Khan, Paul Murray, Carrie Willcox, Tracey Haigh, Benjamin Willcox, Navta Masand, Claire Bowen, Danai B. Dimakou, Jianmin Zuo, Sierra M. Barone, Jonathan M. Irish, Pamela Kearns, and Graham S. Taylor**

# Supplementary Figures and Tables

Table S1

**Paediatric Healthy**

| Study Number | Age | Gender |
|---|---|---|
| HV01 | 37m | Female |
| HV05 | 12m | Male |
| HV06 | 17m | Female |
| HV11 | 19m | Male |
| HV12 | 72m | Female |
| HV13 | 170m | Female |
| HV14 | 20m | Female |
| HV15 | 22m | Male |
| HV16 | 78m | Male |
| HV18 | 15m | Male |
| HV20 | 110m | Female |
| HV21 | 74m | Female |
| HV22 | 42m | Male |
| HV25 | 77m | Male |
| HV26 | 12m | Female |
| HV28 | 35m | Male |
| HV30 | 126m | Male |
| HV34 | 174m | Female |
| HV37 | 54m | Female |

**Paediatric Cancer**

| Study Number | Age | Gender | Disease |
|---|---|---|---|
| C01 | 79m | Female | Rhabdomyosarcoma |
| C02 | 73m | Male | Burkitt's lymphoma |
| C03 | 36m | Female | Neuroblastoma |
| C04 | 167m | Female | Hodgkin's disease |
| C05 | 58m | Male | Wilms |
| C06 | 187m | Female | Rhabdomyosarcoma |
| C07 | 122m | Male | Hodgkin's disease |
| C08 | 20m | Male | Neuroblastoma |
| C09 | 17m | Female | Rhabdoid tumour |
| C10 | 145m | Female | Burkitt's lymphoma |
| C11 | 17m | Male | Neuroblastoma |
| C12 | 22m | Female | Pilomyxoid astrocytoma |
| C14 | 179m | Female | Ewing's sarcoma |
| C15 | 187m | Male | Hodgkin's disease (non classical NHPHL) |
| C16 | 12m | Male | Neuroblastoma |
| C18 | 36m | Female | Hepatoblastoma |
| C19 | 12m | Male | Nephroblastomatosis |
| C20 | 73m | Male | Hodgkin's disease |
| C21 | 34m | Male | Burkitts lymphoma |
| C22 | 75m | Female | Parameningeal Rhabdomyosarcoma |

**Table S1. Demographic data for the paediatric healthy donors and paediatric cancer patients (discovery cohort), related to Figures 1 and 2.** The diagnosis for each patient is also shown. Age is provided in months.

Table S2

| Metal | Marker | Clone | Source |
|---|---|---|---|
| 89Y | CD45 (BC) | H130 | Fluidigm |
| 114 Qdot | CD45 (BC) | H130 | Biolegend |
| 115In | CD45 (BC) | H130 | Biolegend |
| 141Pr | CD31 | WM59 | Biolegend |
| 142Nd | CD57 | HCD57 | Fluidigm |
| 143Nd | CD38 | HIT2 | Biolegend |
| 144Nd | CD8 | SK1 | Biolegend |
| 145Nd | CD4 | RPA-T4 | Fluidigm |
| 146Nd | IgD | IA6-2 | Fluidigm |
| 147Sm | CXCR3 | G025H7 | Biolegend |
| 148Sm | CD16 | 3G8 | Fluidigm |
| 149Sm | CD127 | AO19D5 | Fluidigm |
| 150Nd | OX40 | ACT35 | Fluidigm |
| 151Eu | CCR6 | G034E3 | Biolegend |
| 152Sm | TCR-gd | 11F2 | Fluidigm |
| 153Eu | CCR4 | L291H4 | Biolegend |
| 154Sm | CD73 | AD2 | Biolegend |
| 155Gd | PD1 | EH12.2H7 | Fluidigm |
| 156Gd | CD45RA | HI100 | Biolegend |
| 158Eu | CD33 | WM53 | Fluidigm |
| 159Tb | CD161 | HP-3G10 | Fluidigm |
| 160Gd | CD39 | A1 | Fluidigm |
| 161Dy | ICOS | C398.4A | Biolegend |
| 162Dy | CD27 | L128 | Fluidigm |
| 163Dy | CD56 | NCAM16.2 | Fluidigm |
| 164Dy | CD95 | DX2 | Fluidigm |
| 165Ho | CD19 | HB19 | Fluidigm |
| 166Er | CD24 | ML5 | Fluidigm |
| 167Er | CCR7 | G043H7 | Biolegend |
| 168Er | CXCR5 | J252D4 | Biolegend |
| 169Tm | CD25 | 2A3 | Fluidigm |
| 170Er | CD123 | 6H6 | Biolegend |
| 171Yb | CD5 | UCHT2 | Biolegend |
| 172Yb | CD11c | 3.9. | Biolegend |
| 173Yb | CD3 | UCHT1 | Biolegend |
| 174Yb | HLA-DR | L243 | Fluidigm |
| 175Lu | CD14 | M5E2 | Fluidigm |
| 176Yb | TCR va7.2 | 3C10 | Biolegend |
| 194-198Pt | CD45 (BC) | H130 | Biolegend |
| 209Bi | CD11b | ICRF44 | Fluidigm |

**Table S2. Mass cytometry antibody panel used to characterise the immune cell phenotype of the discovery cohort, related to Figures 1 and 2.** CD45-specific antibodies used for barcoding are indicated with (BC) in the table.

Table S3

| Metal | Marker | Clone | Source |
|---|---|---|---|
| 89Y | | | |
| 114 Qdot | | | |
| 115In | CD45 (BC) | H130 | Biolegend |
| 141Pr | gamma-delta 2 | 123R3 | Milteny |
| 142Nd | CD57 | HCD57 | Fluidigm |
| 143Nd | | | |
| 144Nd | CD8 | SK1 | Biolegend |
| 145Nd | CD4 | RPA-T4 | Fluidigm |
| 146Nd | | | |
| 147Sm | CXCR3 | G025H7 | Biolegend |
| 148Sm | CD16 | 3G8 | Fluidigm |
| 149Sm | CD127 | AO19D5 | Fluidigm |
| 150Nd | | | |
| 151Eu | CCR6 | G034E3 | Biolegend |
| 152Sm | CD27 | M-T271 | |
| 153Eu | CX3CR1 | 2A91 | Biolegend |
| 154Sm | TIGIT | A15153G | Biolegend |
| 155Gd | PD1 | EH12.2H7 | Fluidigm |
| 156Gd | CD45RA | HI100 | Biolegend |
| 158Eu | | | |
| 159Tb | Nkp30 | Z25 | Fluidigm |
| 160Gd | gamma-delta 1 | REA173 | Milteny |
| 161Dy | | | |
| 162Dy | Nkp46 | BAB281 | Fluidigm |
| 163Dy | CD56 | NCAM16.2 | |
| 164Dy | CD95 | DX2 | |
| 165Ho | 1° NKG2C-PE<br>2° Anti-PE | 134591,<br>PE2 | R&D Biosystems,<br>Fluidigm |
| 166Er | NKG2D | ON72 | Fluidigm |
| 167Er | CCR7 | G043H7 | Biolegend |
| 168Er | CXCR5 | J252D4 | Biolegend |
| 169Tm | NKG2A | Z199 | Fluidigm |
| 170Er | CD161 | HP3610 | Biolegend |
| 171Yb | DNAM | DX11 | Fluidigm |
| 172Yb | | | |
| 173Yb | CD3 | UCHT1 | Biolegend |
| 174Yb | | | |
| 175Lu | CD14 | M5E2 | Fluidigm |
| 176Yb | TCR-va 7.2 | 3C10 | Biolegend |
| 194Pt | CD45 (BC) | H130 | Biolegend |
| 198Pt | CD45 (BC) | H130 | Biolegend |
| 209Bi | CD11b | ICRF44 | Fluidigm |

**Table S3. Mass cytometry antibody panel used to characterise NK cell phenotype (discovery cohort), related to Figure 3**. CD45-specific antibodies used for barcoding are indicated with (BC) in the table.

Table S4

| Fluorochrome | Antibody | Clone | Source |
|---|---|---|---|
| e450 | CD3 | SK7 | eBioscience |
| APC | CD56 | CMSSB | eBioscience |
| PeCy7 | CD16 | eBioCB16 | eBioscience |
| PerCP-Cy5.5 | CD57 | QA17A04 | Biolegend |
| FITC | CD107a | H4A3 | Biolegend |
| AF700 | TNF-a | Mab11 | Biolegend |
| PE | INF-g | 4S.B3 | Biolegend |
| APC-Cy7 | CD19 | HIB19 | eBioscience |
| APC-Cy7 | CD14 | 6ID3 | eBioscience |
| APC-Cy7* | Live-Dead | | Biolegend |
| PeCy7 | perforin | dG9 | eBioscience |
| PE-texas red | granzyme b | GB11 | Invitrogen |

**Table S4. Fluorescent flow cytometry antibody panel used for further NK cell characterisation, related to Figure 3.** *e-Fluor 780 live dead stain detected on APC-CY7 detector.

Table S5

**Paediatric Healthy**

| Study Number | Age | Gender |
|---|---|---|
| HV04 | 16m | Female |
| HV07 | 7m | Male |
| HV11 | 19m | Male |
| HV12 | 72m | Female |
| HV13 | 170m | Female |
| HV14 | 20m | Female |
| HV15 | 22m | Male |
| HV16 | 78m | Male |
| HV19 | 51m | Male |
| HV21 | 74m | Female |
| HV23 | 30m | Male |
| HV24 | 137m | Female |
| HV25 | 77m | Male |
| HV27 | 109m | Male |
| HV30 | 126m | Male |
| HV31 | 11m | Male |
| HV37 | 54m | Female |
| HV38 | 153m | Male |
| HV39 | 147m | Female |
| HV40 | 155m | Male |
| HV41 | 154m | Male |
| HV42 | 150m | Male |
| HV43 | 155m | Male |
| HV44 | 124m | Male |

**Paediatric Cancer**

| Study Number | Age | Gender | Disease |
|---|---|---|---|
| C23 | 56m | Female | Wilms |
| C24 | 172m | Female | Hodgkins Disease |
| C25 | 29m | Male | Neuroblastoma |
| C26 | 167m | Female | Osteosarcoma |
| C27 | 179m | Male | Hodgkins Disease |
| C28 | 59m | Male | Rhabdomyosarcoma |
| C29 | 51m | Male | Rhabdomyosarcoma |
| C30 | 189m | Female | Ewings sarcoma |
| C31 | 176m | Male | Hodgkins Disease |
| C32 | 118m | Male | Hodgkins Disease |
| C33 | 130m | Female | Ewings sarcoma |
| C34 | 47m | Female | Wilms |
| C35 | 183m | Male | Osteosarcoma |
| C36 | 19m | Female | Wilms |
| C37 | 38m | Female | Wilms |
| C38 | 32m | Male | Embryonal supratentorial tumour |
| C39 | 143m | Male | Osteosarcoma |
| C40 | 163m | Female | Hodgkins Disease |
| C41 | 162m | Male | Mixed Germ Cell Tumour |
| C42 | 149m | Male | Rhabdomyosarcoma |
| C44 | 87m | Male | B-cell Lymphoma |
| C45 | 189m | Male | Hodgkins Disease |
| C46 | 86m | Male | Rhabdomyosarcoma |
| C47 | 4m | Female | Rhabdoid tumour |
| C48 | 170m | Male | Hodgkins disease |
| C49 | 188m | Female | Rhabdoid tumour |
| C50 | 88m | Female | Hepatoblastoma |
| C51 | 65m | Male | Rhabdomyosarcoma |
| C52 | 152m | Male | Rhabdomyosarcoma |
| C53 | 158m | Female | Osteosarcoma |
| C54 | 169m | Female | Ewings sarcoma |

**Table S5. Demographic data for the paediatric healthy donors and paediatric cancer patients (validation cohort), related to Figures 4 and 5.** The diagnosis for each patient is also shown. Age is provided in months.

Table S6

| Metal | Marker | Clone | Source | Metal | Marker | Clone | Source |
|---|---|---|---|---|---|---|---|
| 89Y | CD45 (BC) | HI30 | Fluidigm | 161Dy | ILT3 | ZM4.1 | Biolegend |
| 106Cd | CD45 (BC) | HI30 | Biolegend | 161Dy | ILT5 | 222821 | R&D Systems |
| 110Cd | CD45 (BC) | HI30 | Biolegend | 161Dy | ILT4 | 42D1 | Fluidigm |
| 111Cd | CD38 | HIT2 | Biolegend | 162Dy | NKp44 | P44-8 | Biolegend |
| 112Cd | CCR2 | K036C2 | Biolegend | 162Dy | NKp46 | BAB281 | Fluidigm |
| 114Cd | CD8a | SK1 | Biolegend | 163Dy | CXCR4 | 12G5 | Biolegend |
| 115In | CD57 | HNK-1 | Biolegend | 163Dy | CXCR3 | G025H7 | Fluidigm |
| 116Cd | CD36 | 5-271 | Biolegend | 164Dy | CD161 | HP3G10 | Fluidigm |
| 141Pr | CD3 | UCHT1 | Fluidigm | 165Ho | KIR2DS4 | 179315 | R&D Systems |
| 142Nd | CD19 | HIB19 | Fluidigm | 166Er | NKG2D | ON72 | Biolegend |
| 143Nd | CD45RA | HI100 | Fluidigm | 167Er | KIR3DL1 | DX9 | Fluidigm |
| 144Nd | CD69 | FN50 | Fluidigm | 167Er | KIR3DL2 | 539304 | R&D Systems |
| 145Nd | CD4 | RPA-T4 | Fluidigm | 168Er | CD127 | A019D5 | Fluidigm |
| 146Nd | KIR2DL1/S1/S3/S5 | HP-MA4 | Biolegend | 169Tm | NKG2A | Z199 | Fluidigm |
| 147Sm | CXCR1 | 42705 | R&D Systems | 170Er | CD122 | Tu27 | Fluidigm |
| 147Sm | CXCR2 | 5E8/CXCR2 | Fluidigm | 171Yb | CD226 | DX11 | Fluidigm |
| 148Nd | CD14 | RMO52 | Fluidigm | 172Yb | CX3CR1 | 2A9-1 | Fluidigm |
| 149Sm | CD25 | 2A3 | Fluidigm | 173Yb | KIR2DL2/L3 | DX27 | Fluidigm |
| 150Nd | CD27 | LG.3A10 | Fluidigm | 173Yb | KIR2DL5 | UP-R1 | Miltenyi Biotec |
| 151Eu | KIR2DL1/S5 | 143211 | R&D Systems | 174Yb | CD94 | HP-3D9 | Fluidigm |
| 152Sm | ILT1 | 337902 | Biolegend | 175Lu | PD-1 | EH12.2H7 | Fluidigm |
| 153Eu | CXCR5 | RF8B2 | Fluidigm | 176Yb | KIR2DL4 | 181703 | R&D Systems |
| 154Sm | TIGIT | MBSA43 | Fluidigm | 194Pt | CD45 (BC) | HI30 | Biolegend |
| 154Sm | TIM3 | F38-2E2 | Fluidigm | 195Pt | CD45 (BC) | HI30 | Biolegend |
| 155Gd | CD56 | B159 | Fluidigm | 196Pt | CD45 (BC) | HI30 | Biolegend |
| 156Gd | ILT2 | GHI/75 | Fluidigm | 198Pt | KIR2DL2/L3/S2/S4 | 180704 | R&D Systems |
| 158Gd | ILT2/LIR6 | 586326 | R&D Systems | 209Bi | CD16 | 3G8 | Fluidigm |
| 159Tb | NKp80/NKp30 | 239127/Z25 | R&D Systems/Fluidigm | | | | |
| 160Gd | CXCR6 | K041E5 | Fluidigm | | | | |

**Table S6. Mass cytometry antibody panel used to characterise the Validation Cohort, related to Figures 4 and 5.** CD45-specific antibodies used for barcoding are indicated with (BC) in the table.

Table S7

| Study Number | Lymphocytes x10^9/L | NK cells % from total lymphocytes | Absolute Count of NK cells |
|---|---|---|---|
| C23 | 2 | 11.984 | 0.239674 |
| C24 | 1.9 | 9.1177 | 0.173236 |
| C25 | 3.5 | 7.2734 | 0.254569 |
| C26 | N/A | 2.944 | N/A |
| C27 | 2.2 | 11.118 | 0.244592 |
| C28 | 2.5 | 2.0261 | 0.050653 |
| C29 | 3.3 | 6.9097 | 0.22802 |
| C30 | 2 | 6.6499 | 0.132998 |
| C31 | 2 | 5.6369 | 0.112738 |
| C32 | 2.8 | 1.1862 | 0.033214 |
| C33 | 1.2 | 5.5849 | 0.067019 |
| C34 | 2.2 | 3.5674 | 0.078483 |
| C35 | 0.9 | 2.563 | 0.023067 |
| C36 | 6.2 | 9.3255 | 0.578181 |
| C37 | 4.5 | 5.0394 | 0.226773 |

| Study Number | Lymphocytes x10^9/L | NK cells % from total lymphocytes | Absolute Count of NK cells |
|---|---|---|---|
| C38 | 5.5 | 8.6934 | 0.478137 |
| C39 | 2 | 3.6367 | 0.072734 |
| C40 | 0.9 | 8.4769 | 0.076292 |
| C41 | 3 | 6.3382 | 0.190146 |
| C42 | 1.5 | 6.3902 | 0.095853 |
| C44 | 1.8 | 11.689 | 0.210407 |
| C45 | 0.8 | 7.9574 | 0.063659 |
| C46 | 3.5 | 3.6194 | 0.126679 |
| C47 | 6 | 1.5759 | 0.094554 |
| C48 | 1.5 | 24.652 (high) | 0.369773 |
| C49 | 1.3 | 5.6801 | 0.073841 |
| C50 | 1.9 | 4.39 | 0.08341 |
| C51 | 1.6 | 2.8574 | 0.045718 |
| C52 | 2.6 | 5.5676 | 0.144758 |
| C53 | 2.1 | 7.7496 | 0.162742 |
| C54 | 3.3 | 6.9703 | 0.23002 |

**Table S7. Lymphocyte count, NK cell frequency and absolute count of NK cells for patients C23-C54 (validation cohort), related to Figures 4 and 5.** Values below the normal range for each child are shown in red font. Note that the normal range for lymphocytes and NK cells in children varies with age. The normal range appropriate for each child's age has been used to determine which values are abnormal.

Figure S1

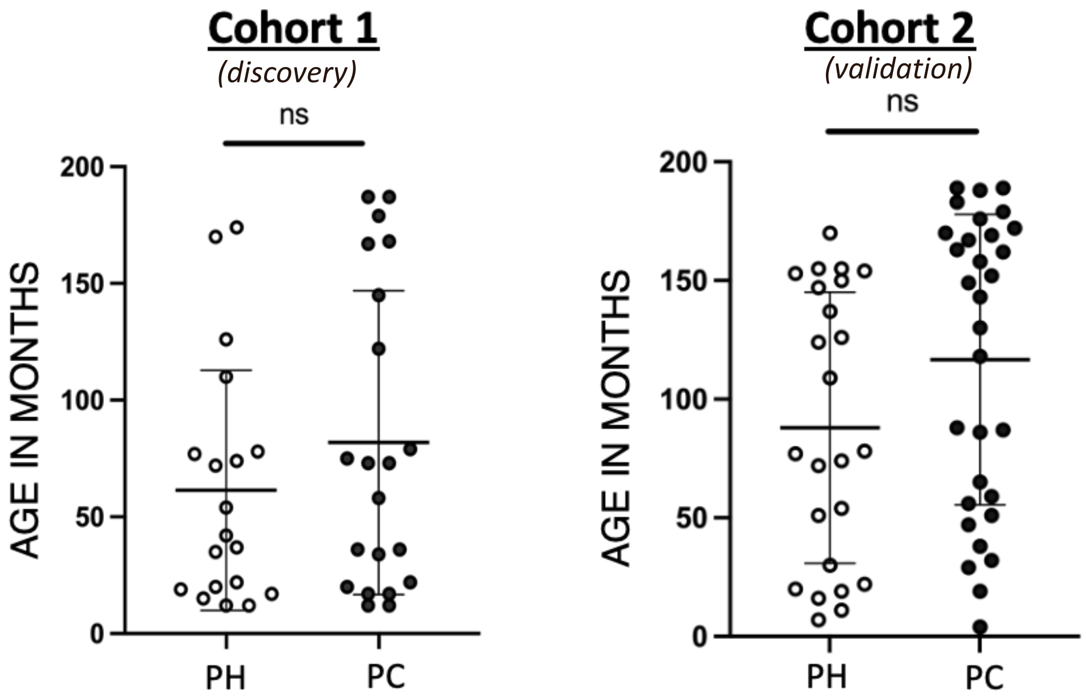


**Figure S1. Age distribution of paediatric healthy (PH) or paediatric cancer (PC) donors, related to Figures 1-6.** Error bars show mean +/-1 standard deviation. There was no significant difference in age between the two groups (unpaired t-test).

Figure S2

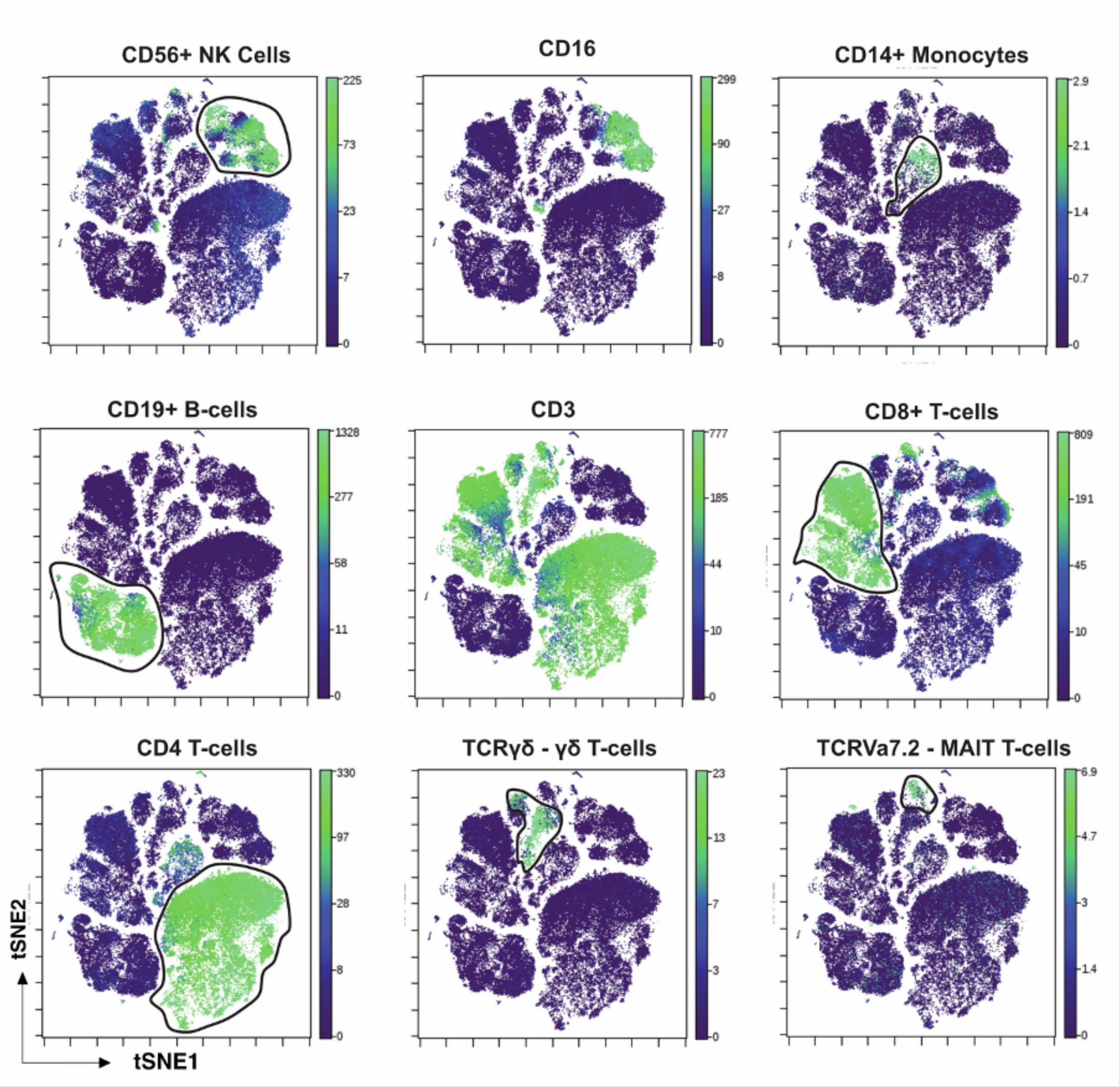


**Figure S2. Gating strategy used to separate total immune cells into subsets for further analysis for discovery cohort, related to Figures 1 and 2.** Each subset is determined by the indicated marker. Intensity of staining is represented by colour, with levels shown on the right-hand side of each plot.

Figure S3.

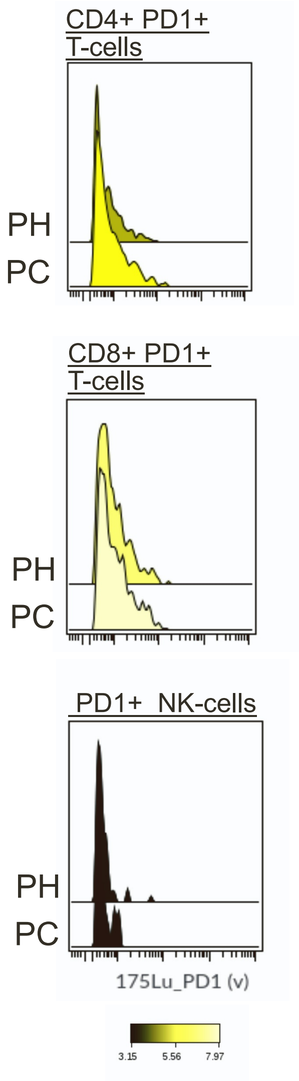


**Figure S3. Median metal intensity (MMI) of PD1 on T-cells and NK cells, related to Figure 2.** Levels of cell surface PD1 for the pediatric healthy and pediatric cancer patients for the discovery cohort are shown**.** For the total cohort the PD1 MMIs for the PD1-positive cells were: CD4 T-cells, mean 5.5, median 5.1 (range 3.8-11.8); CD8 T-cells, mean 8.4, median 7.8 (range 5.5-14.9); NK-cells, mean 3.1, median 3.0 (range 2.5-4.3).

Figure S4

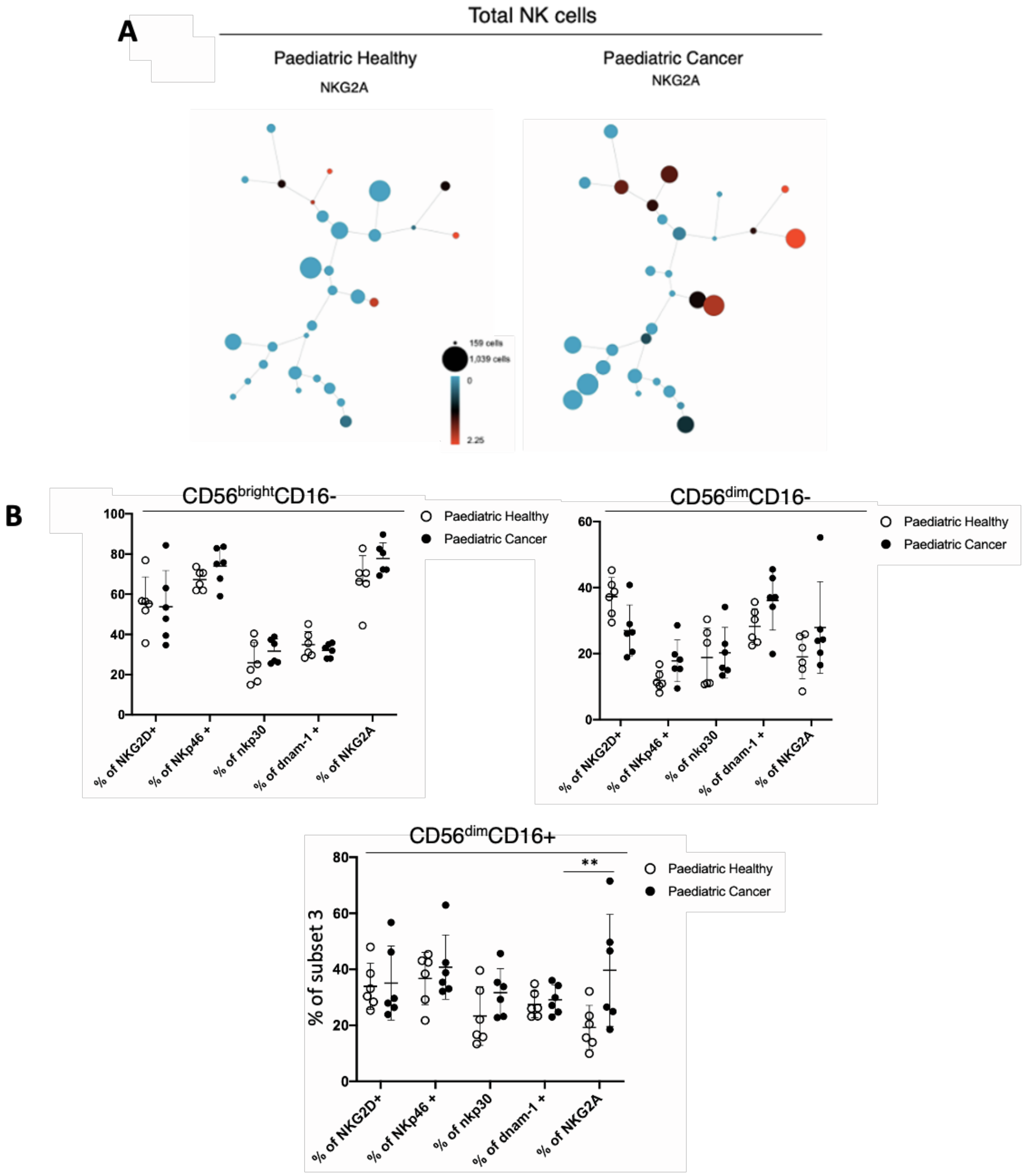


**Figure S4. Analysis of NK cell receptors, related to Figure 3.** A) Total NK cells were clustered using the SPADE algorithm in Cytobank. Spade nodes are coloured by the level of NKG2A expression. Size of each node reflects the numbers of cells. B) Frequency of NK cells within each of four NK cell subsets (defined using CD16 and CD56) showing the percentage of cells positive for each NK receptor. The error bars show the mean +/- one SD. Unpaired t-tests, correcting for multiple comparisons with 5% FDR, were performed with significant results indicated by an asterisk: * p<0.05, ** p<0.01, *** p<0.001.

Figure S5

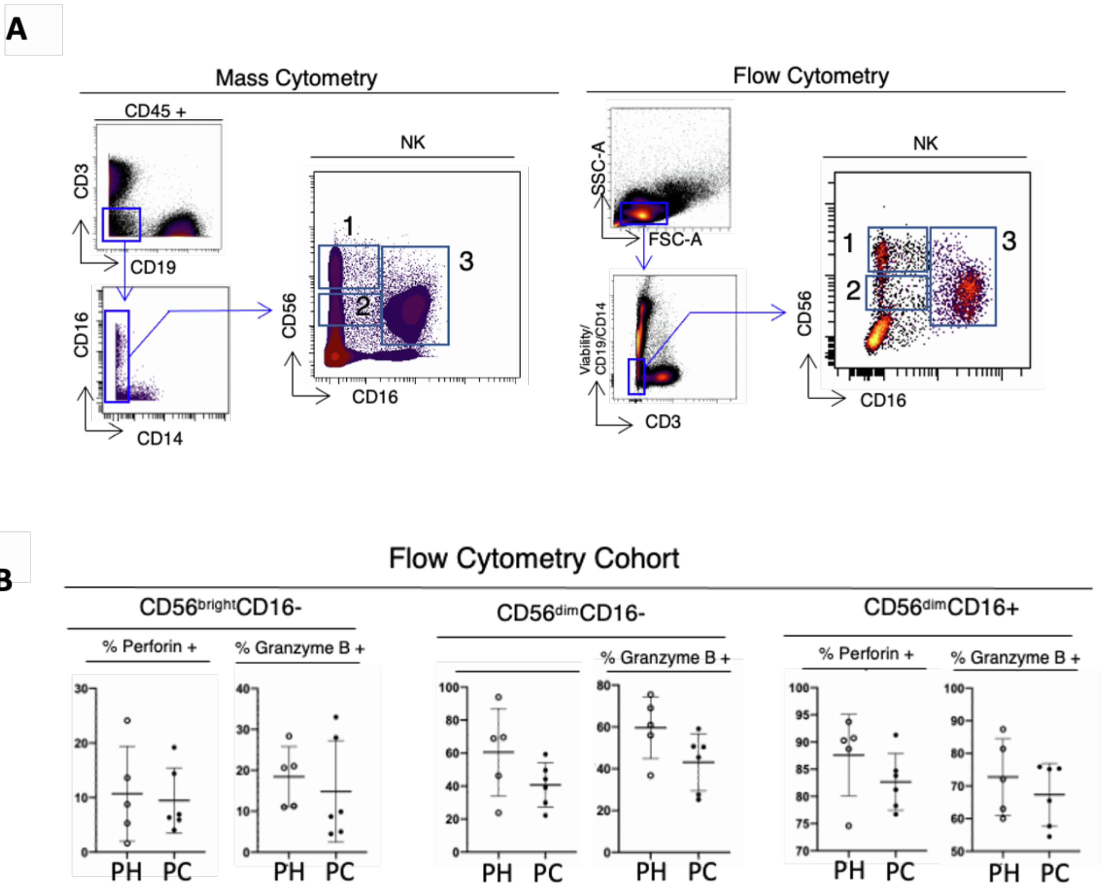


**Figure S5. Perforin and granzyme B expression in NK cells, related to Figure 3.** A) Gating strategy used to investigate the three NK cell subsets (defined using CD16 and CD56) from mass and flow cytometry data. B) Frequency of NK cells within each NK subset showing the percentage of cells positive for perforin or Granzyme B. The error bars show the mean +/- one SD. Unpaired t-test, correcting for multiple comparisons with 5% FDR, were performed with no significant results identified.

Figure S6

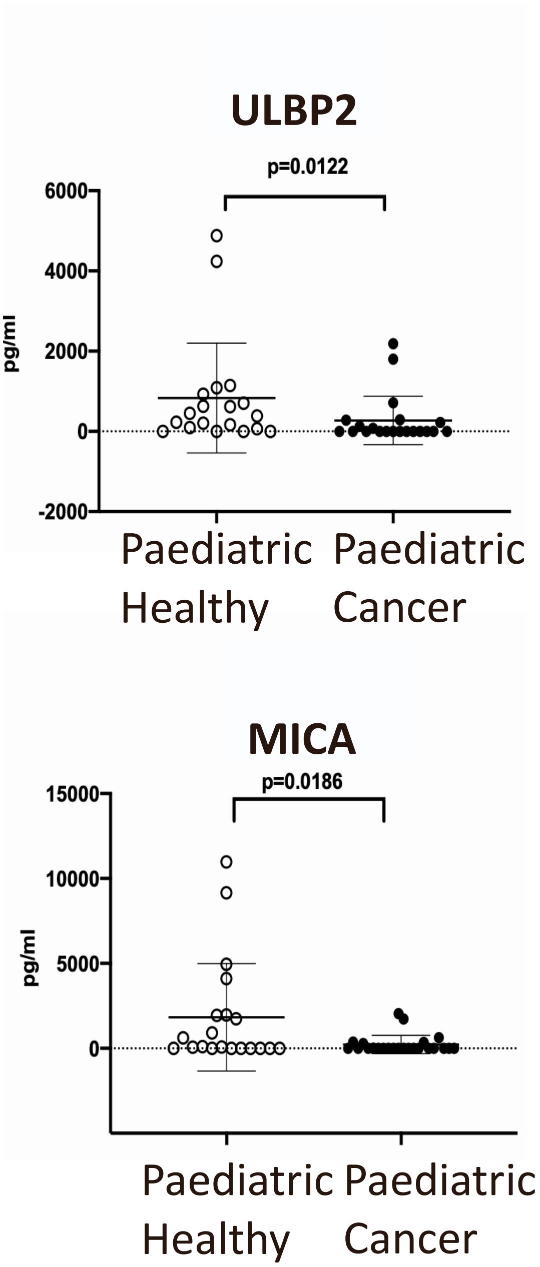


**Figure S6. Results of ELISAs measuring NKG2D ligands ULBP2 or MICA in plasma, related to Figure 3.** The error bars show the mean +/- one SD. Mann-Whitney tests were used to a compare paediatric healthy and paediatric cancer patients (ULBP-2, p=0.0122; MICA, p=0.0186).

Figure S7

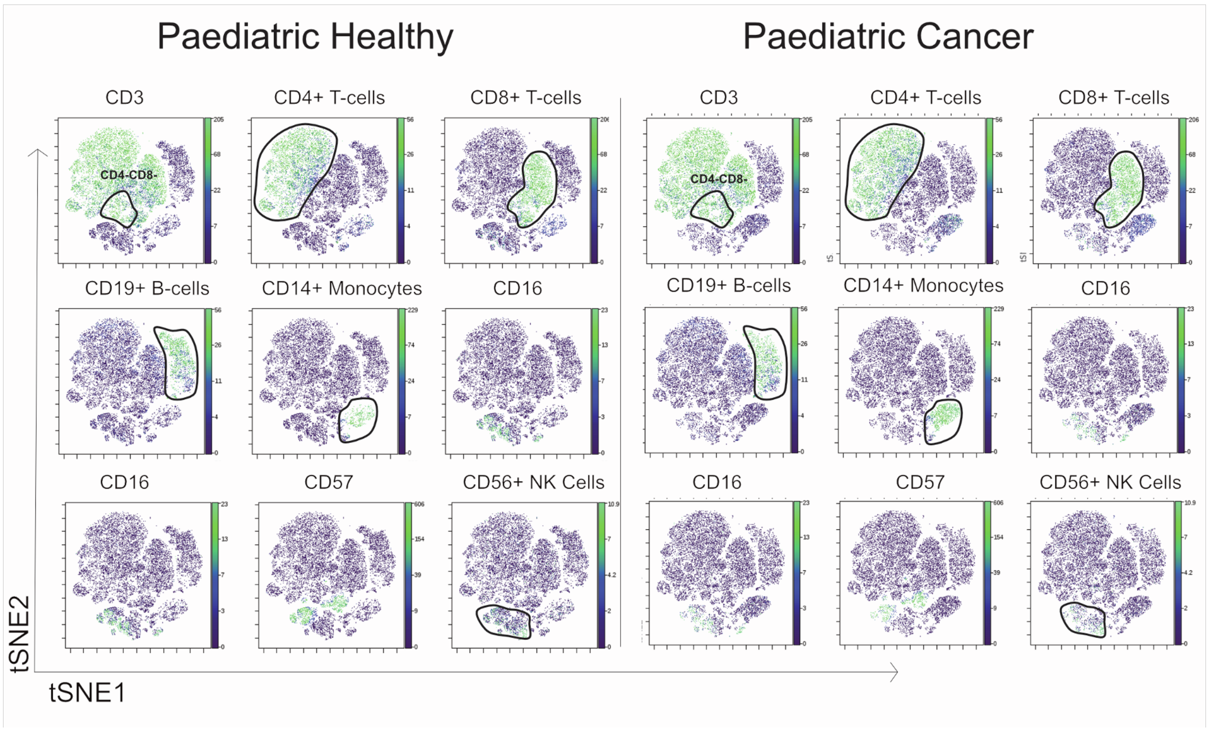


**Figure S7. Gating strategy used to separate total immune cells into subsets for further analysis, related to Figure 4.** Each subset is determined by the indicated marker. Intensity of staining is represented by colour, with levels shown on the right-hand side of each plot.

Figure S8

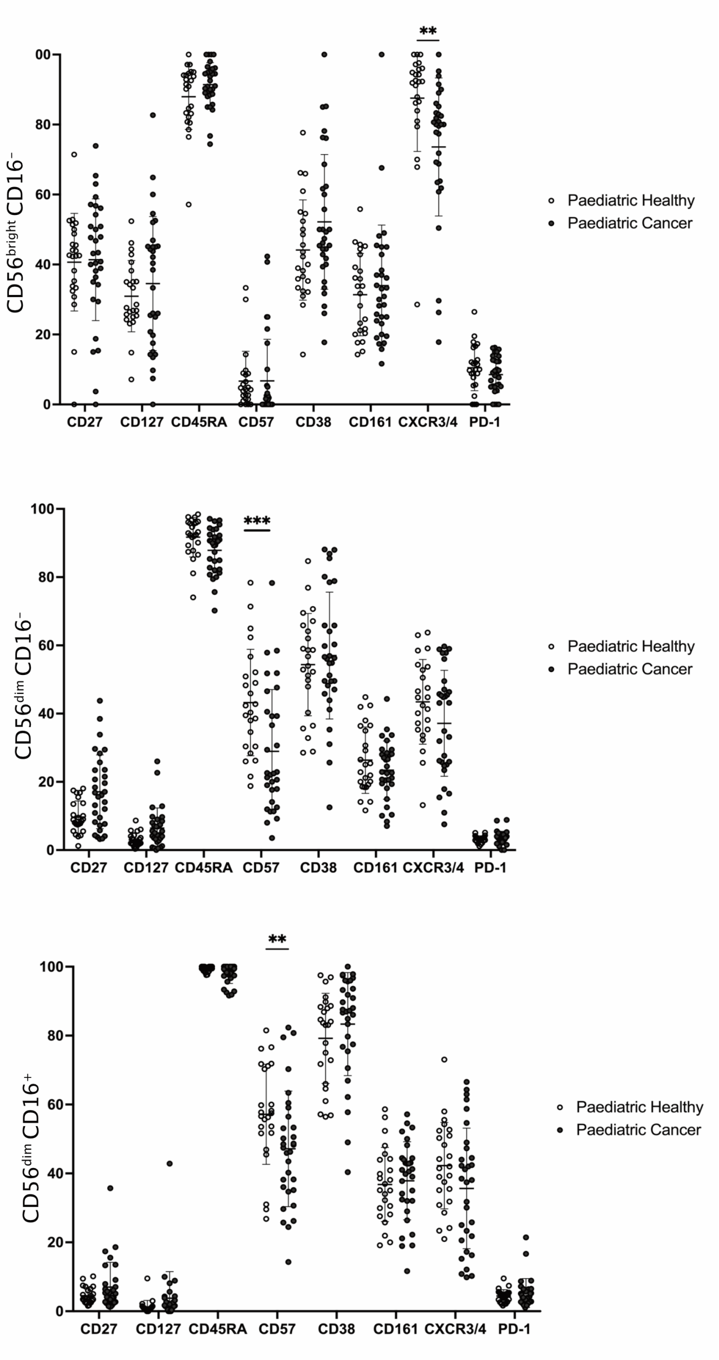


**Figure S8. Percentage of cells within each NK cell subset positive for cell surface molecules, related to Figure 4.** The error bars show the mean +/- one standard variation. P values were calculated using Wilcoxon ranked sum tests with false discovery rate at 5% and correction using the Benjamini-Hochberg method. Significant results are indicated: * p<0.05, ** p<0.01, *** p<0.001.

Figure S9

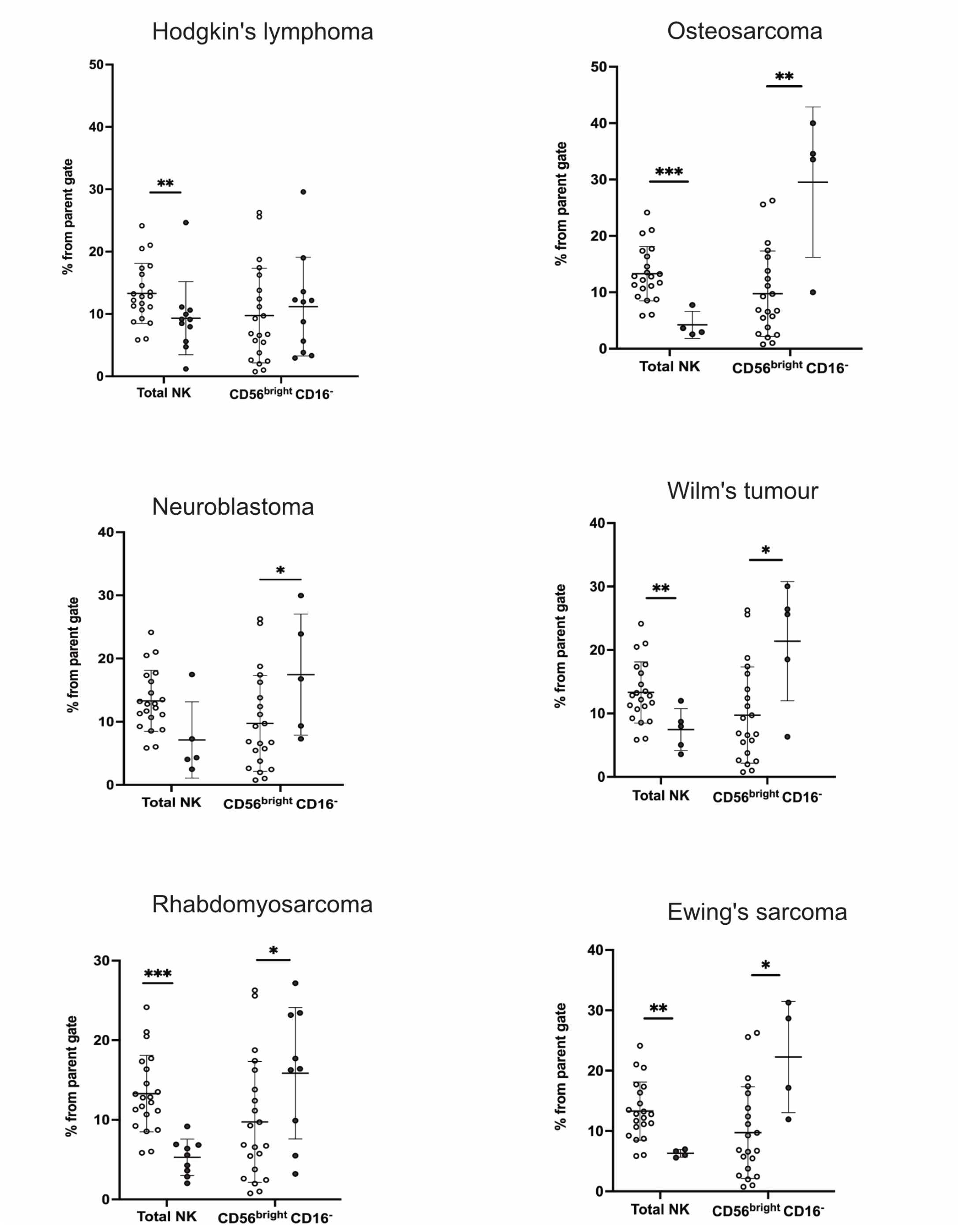


**Figure S9. NK cell frequencies in patients with different cancer types, related to Figures 4 and 5.** Total NK cell and CD56*bright*CD16- NK cells were analysed for each individual for the six most common paediatric cancers as shown. The error bars show the mean +/- one standard variation. P values were calculated using Wilcoxon ranked sum tests with false discovery rate set at 5% and correction using the Benjamini-Hochberg method. Significant results are indicated as: * $p<0.05$, ** $p<0.01$, *** $p<0.001$

Figure S10

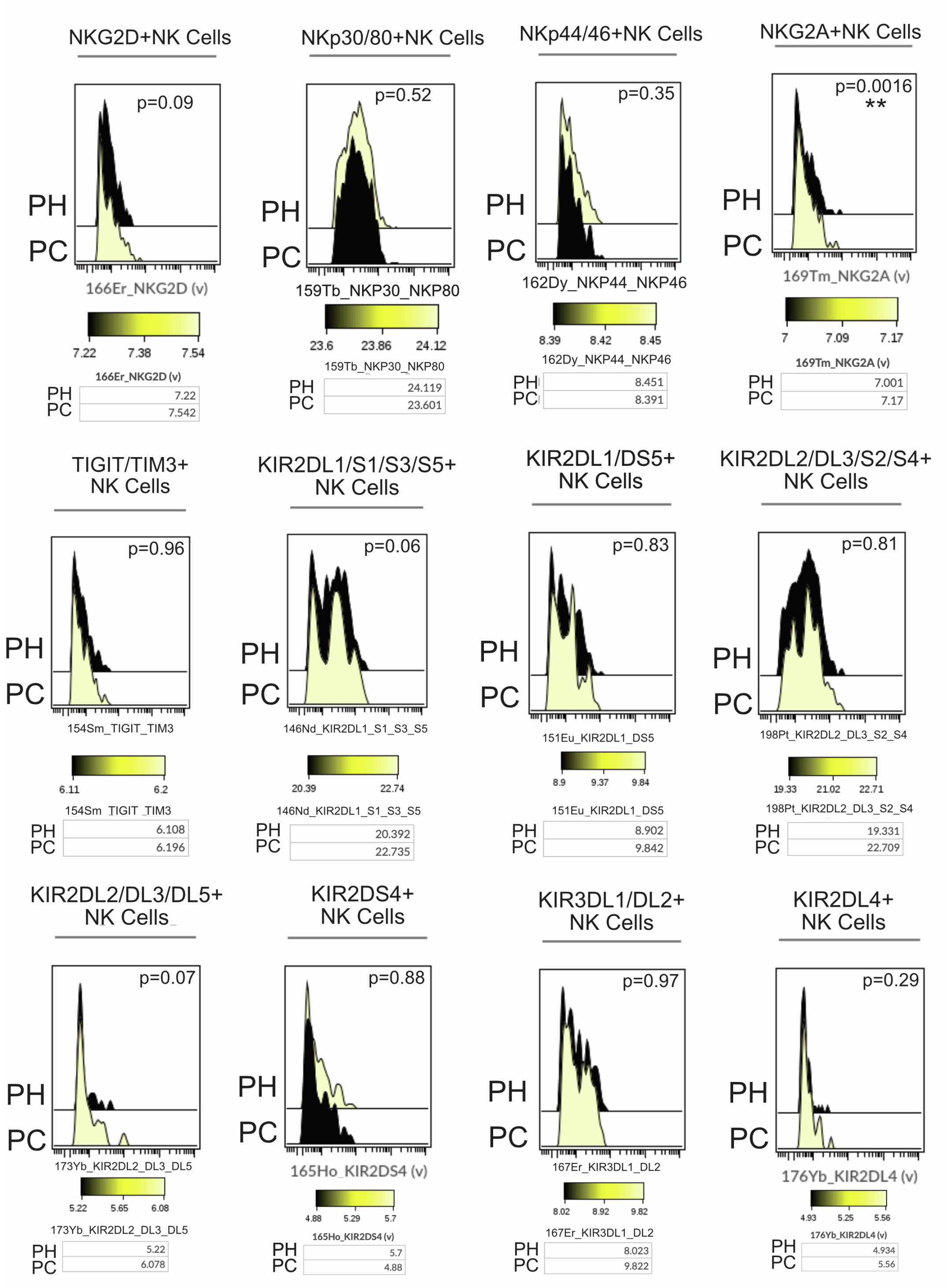


**Figure S10. Median metal intensity (MMI) of NK cell receptors, related to Figure 5.** The median metal intensity of each marker on NK cells gated within the positive gate for that marker are shown. P values were calculated using Wilcoxon ranked sum tests with false discovery rate at 5% and correction using the Benjamini-Hochberg method. Significant results are indicated: * p<0.05, ** p<0.01, *** p<0.001.

Figure S11

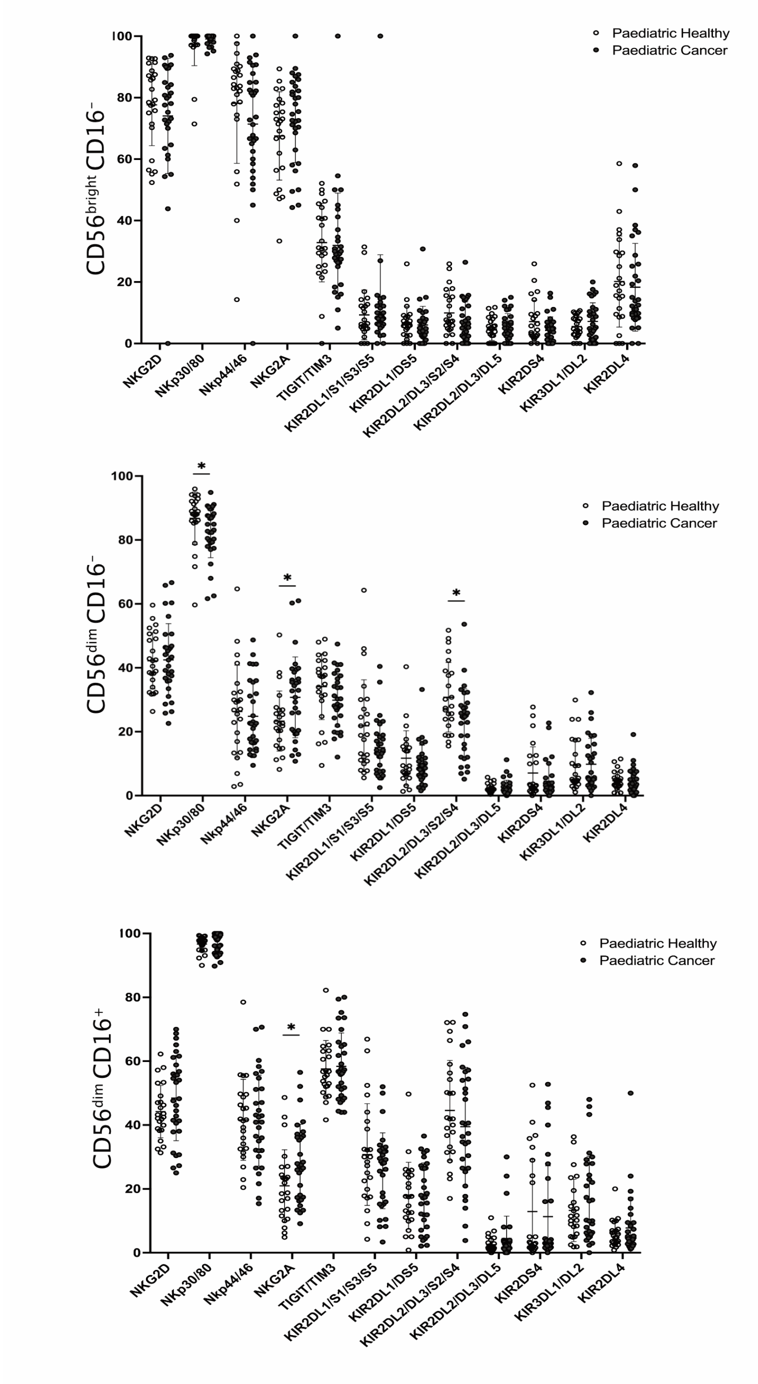


**Figure S11. NK cell receptor expression for each NK subset. Values are shown for each individual, related to Figure 5.** The error bars show the mean +/- one standard variation. P values were calculated using Wilcoxon ranked sum tests and are shown in the figure. When we applied a false discovery rate set at 5% and used the Benjamini-Hochberg multiple comparison correction method no statistical significant results were identified. Significant results indicated as: * p<0.05, ** p<0.01, *** p<0.001 are before multiple comparison correction.
